# Multi-Omics Analysis of MCM2 as a Promising Biomarker in Pan-Cancer

**DOI:** 10.3389/fcell.2022.852135

**Published:** 2022-05-25

**Authors:** Jing Yuan, Hua Lan, Dongqing Huang, Xiaohui Guo, Chu Liu, Shuping Liu, Peng Zhang, Yan Cheng, Songshu Xiao

**Affiliations:** ^1^ Department of Gynecology and Obstetrics, Third Xiangya Hospital, Central South University, Changsha, China; ^2^ Department of Gynecology, The Second Hospital of Zhuzhou, Zhuzhou, China; ^3^ Department of Rehabilitation, Changsha Central Hospital of University of South China, Changsha, China; ^4^ Graduate Collaborative Training Base of the Affiliated Nanhua Hospital, Hengyang Medical School, University of South China, Hengyang, China; ^5^ Department of Pharmacy, The Second Xiangya Hospital, Central South University, Changsha, China

**Keywords:** MCM2, comprehensive bioinformatics, immune therapy response, prognostic value, skin cutaneous melanoma

## Abstract

Minichromosome maintenance 2 (MCM2) is a member of the minichromosomal maintenance family of proteins that mainly regulates DNA replication and the cell cycle and is involved in regulating cancer cell proliferation in various cancers. Previous studies have reported that MCM2 plays a pivotal role in cell proliferation and cancer development. However, few articles have systematically reported the pathogenic roles of MCM2 across cancers. Therefore, the present pan-cancer study was conducted. Various computational tools were used to investigate the MCM2 expression level, genetic mutation rate, and regulating mechanism, immune infiltration, tumor diagnosis and prognosis, therapeutic response and drug sensitivity of various cancers. The expression and function of MCM2 were examined by Western blotting and CCK-8 assays. MCM2 was significantly upregulated in almost all cancers and cancer subtypes in The Cancer Genome Atlas and was closely associated with tumor mutation burden, tumor stage, and immune therapy response. Upregulation of MCM2 expression may be correlated with a high level of alterations rate. MCM2 expression was associated with the infiltration of various immune cells and molecules and markedly associated with a poor prognosis. Western blotting and CCK-8 assays revealed that MCM2 expression was significantly upregulated in melanoma cell lines. Our results also suggested that MCM2 promotes cell proliferation *in vitro* by activating cell proliferation pathways such as the Akt signaling pathways. This study explored the oncogenic role of MCM2 across cancers, provided data on the underlying mechanisms of these cancers for further research and demonstrated that MCM2 may be a promising target for cancer immunotherapy.

## Introduction

The minichromosomal maintenance (MCM) family is a group of protein-coding genes whose products participate in the assembly of a pre-replicative complex (pre-RC) that combines with the origin recognition complex (ORC) and Cdc6 and Cdt1 to regulate DNA replication (Champeris Tsaniras et al., 2014; Fragkos et al., 2015) and cell cycle transition, affecting proliferation (Li and Xu, 2019). Generally, MCM2–7 always connect to each other to form a complex with a heterohexameric structure, which acts as a DNA replicative helicase and AAA + ATPase in the initiation and elongation stage of DNA replication (Tye, 1999; Labib et al., 2000). MCM8–9 also form a complex that is mainly present in vertebrates (Liu et al., 2009). MCM8–9 may play a role in homologous recombination (HR) repair and are required for cisplatin resistance (Nishimura et al., 2012; Park et al., 2013; Lee et al., 2015). Several decades of research have determined that MCM10 serves as a scaffold protein by binding to the MCM2–7 complex to form an integral replication component during the regulation of DNA replication (Brosh and Trakselis, 2019). Recent studies have revealed the importance of MCMs for regulating cancer cell proliferation and serving as biomarkers for diagnosis, prognosis and therapy, such as MCM8 in bladder cancer (Zhu et al., 2021). In addition, studies have also reported that gene mutations in MCMs have been linked to immunodeficiency. For example, a mutation in MCM4 in cancer leads to significantly decreased levels of natural killer cells, indicating that some MCMs may act as regulators of the immune system and contribute to human responses to immune therapy (Gineau et al., 2012).

MCM2 is a member of the MCM family and plays an essential role in the formation of the replication initiation complex (Guerrero-Puigdevall et al., 2021). It encodes a protein that has 904 amino acids and a molecular mass of 101,896 Da (Bleichert et al., 2017). MCM2 unwinds DNA, taking part in the initiation of DNA replication by directly binding to DNA replication origins and regulating gene expression (Kearsey et al., 1996; Lei and Tye, 2001). Of all the MCMs, MCM2 is the most researched protein in cancer, which makes it a promising biomarker for diagnosing cancers. MCM2 is highly expressed in solid tumors and silenced in normal samples and a possible prognostic marker and therapeutic target in a group of cancers. For example, MCM2 was highly expressed in tissue samples of lung cancer (Tan et al., 2001) and ovarian cancer (Aihemaiti et al., 2018). Deng et al. (2019) reported that knockdown of MCM2 significantly improved the chemoresistance of ovarian cancer to carboplatin and olaparib, indicating that MCM2 may be a promising therapeutic target (Zhong et al., 2018). Recently, MCM2 has been identified as a vital downstream molecule of various oncogenes, such as CAMKK2 and MEK1 (Najar et al., 2021).

However, according to previous studies, MCM2 plays an role in promoting cancer development in different cancer subtypes. This paper is the first to explore the expression and biofunction of MCM2 from the perspective of various cancers, focusing on its diagnostic and prognostic values to provide a more systematic and comprehensive insight into MCM2. We found that MCM2 is not only significantly upregulated in 33 types of human cancers but also differentially expressed in the different immune subtypes and molecular subtypes of eight cancers. Additionally, MCM2 is high accurate in diagnosing various cancers. Next, we investigated the mutation rate and the relationship between MCM2 expression and the infiltration of immune-related cells and molecules across cancers. These results may help to identify patients who will have a relatively good response to and prognosis after immune therapy. Moreover, we further investigated the protein–protein interaction (PPI) network correlated with MCM2 and its pathways through GO and KEGG analysis, and GSEA. Finally, we emphasized skin cutaneous melanoma (SKCM) and examined the function of MCM2 in SKCM cell lines by performing cell proliferation experiments. Taken together, our results indicate that MCM2 is a potential biomarker for diagnosis and prognosis across cancers and a promising molecular target for treating SKCM. In this article, we systematically provide the oncogenic role and possible mechanism of MCM2 across cancers through a combined analysis of genomics, transcriptomics and proteomics to explore the potential diagnostic, prognostic and therapeutic value of MCM2 in various cancers.

## Materials and Methods

### mRNA and Protein Levels of MCM2 in Public Databases

**FIGURE 1:**
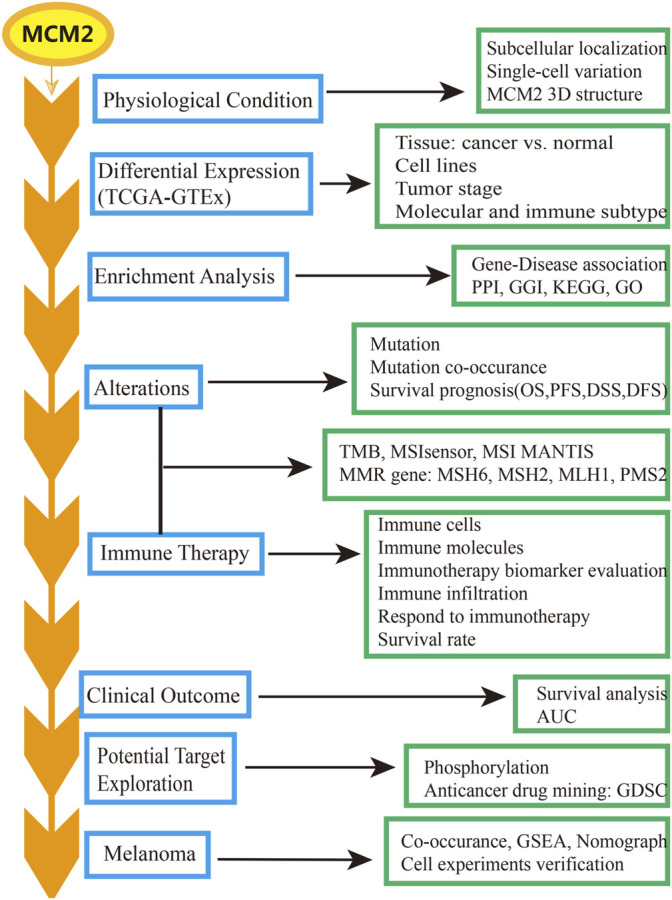
The workflow of the study.

The transcription level of MCM2 across cancers was analyzed by using the ggplot2 package of R version 3.6.3, which analyses data downloaded from TCGA for expression data in cancer samples and data downloaded from GTEx for expression data in normal tissues (Vivian et al., 2017). The Human Protein Atlas (HPA) (www.proteinatlas.org) was used to validate the protein levels of MCM2 (HPA031496) based on an immunohistochemistry platform across cancers (Asplund et al., 2012), the transcription level of MCM2 in normal tissues and cell lines and the localization of MCM2. The workflow of this study is shown in Figure 1 (Wu et al., 2021; Zhang et al., 2021a; Mi et al., 2022). We explored the correlations between MCM2 expression and molecular subtypes or immune subtypes across cancers from the TISIDB database (http://cis.hku.hk/TISIDB), which integrates multiple data types to assess tumor and immune system interactions (Ru et al., 2019). We also explored the correlations between MCM2 expression and immunomodulators across cancers from the TISIDB database.

### Genomic Alteration and Transcriptional Modification Analysis of MCM2

The cBioPortal for cancer genomics database (http://www.cbioportal.org/) has a large number of cancer genomics datasets (Cerami et al., 2012). We used this tool to analyze the different mutation rates of MCM2 across cancers, the mutation sites in amino acids of MCM2, tumor mutation burden (TMB) in MCM2 nonmutant cancer and different subtypes of MCM2 mutant cancer and download the 3D structure of MCM2 (accession number, 6rax). Each gray dot represents one patient. The black line represents the median TMB and its interquartile ranges. The catalog of somatic mutations in cancer (COSMIC) database (https://cancer.sanger.ac.uk/cosmic/) provides data on coding mutations, noncoding mutations genome rearrangements, fusion genes, etc., in the human genome, and was also used to investigate the rate of different types of MCM2 mutations in different cancers.

### Tumor Immune Infiltration Analysis

The Tumor Immune Estimation Resource (TIMER) (https://cistrome.shinyapps.io/timer/) is a comprehensive and public database that includes 10,897 samples of 32 cancers from TCGA (Li et al., 2020). TIMER provides the infiltration of six kinds of immune cells (B cells, CD4^+^ T cells, CD8^+^ T cells, neutrophils, macrophages and dendritic cells). In addition, we examined the correlation between the expression levels of MCM2 and the infiltration levels of immune cells and immune molecules (CD274, CTLA4 and PDCD1) through TIMER. Tumor Immune Dysfunction and Exclusion (TIDE) (http://tide.dfci.harvard.edu/) database was used to speculate the tumor immunity regulated by genes, and to comprehensively analyze the immune escape mechanism of immune dysfunction and rejection on tumors, so as to effectively predict the effect of immune checkpoint inhibition therapy.

### Functional and Pathway Enrichment Analysis

The Gene Multiple Association Network Integration Algorithm GeneMANIA website (www.genemania.org) was applied to predict the relationship between the MCMs and their functionally similar genes to construct the gene-gene interaction (GGI) network (Warde-Farley et al., 2010). The Search Tool for the Retrieval of Interacting Genes (Szklarczyk et al., 2015) (STRING) (www.string-db.org) is an online tool for constructing PPI networks and searching for the top 50 genes positively correlated with MCM2 in SKCM. By uploading the list of 50 hub genes, we constructed a PPI network between MCM2 and its related proteins. The R ggplot2 package (for visualization) and cluster profiler package (for data analysis) were used to conduct a functional enrichment analysis of gene ontology (GO) and Kyoto Encyclopedia of Genes and Genes (KEGG) between MCM2 and neighboring genes. The latest gene annotation of KEGG pathway was obtained from KEGG rest API (https://www.kegg.jp/kegg/rest/keggapi.html). We used gene set enrichment analysis (GSEA) (http://software.broadinstitute.org/gsea/index.jsp) (version 4.1.0) to explore the top 10 signaling pathways of MCM2 in SKCM. MCM2 expression was categorized as high or low according to the median value from the TCGA database, and then the significant difference in enrichment between the two cohorts was analyzed. The UALCAN database (http://ualcan.path.uab.edu/analysis.html) was used to predict the different expression among phosphorylation sites of MCM2 and the expression among different clinical stages based on data from the Clinical Proteomic Tumor Analysis Consortium (CPTAC) Confirmatory/Discovery dataset.

### Clinical Value Analysis

The progression-free survival and overall survival analysis (OS) of MCM2 across cancers was assessed by using the Kaplan–Meier Plotter (www.kmplot.com) based on gene chip and RNA-Seq data from GEO, EGA, TCGA and other public databases (Győrffy et al., 2013). The University of California Santa Cruz (UCSC) Xena website (xena.ucsc.edu) is an online tool used to retrieve quantification expression data, DNA methylation data and copy number data of MCM2 from TCGA samples of various cancers (Goldman et al., 2020). The ROC package in R studio was used to conduct receiver operating characteristic (ROC) analysis of MCM2 and calculate the areas under the curve (AUCs) to estimate the prognostic ability of MCM2 across cancers. The rms package in R studio was used to construct nomograph based on Cox proportional hazards model. GSCALite (http://bioinfo.life.hust.edu.cn/web/GSCALite/) provides a platform for genomic cancer analysis. GSCA integrates 10,000 genomic datasets of 33 cancers from TCGA and more than 750 small-molecule drugs from GDSC and CTRP (Liu et al., 2018). We analyzed the drug sensitivity of MCM2 in various cancers.

### Cell Culture and Transfection and *In Vitro* Knockdown of MCM2

The human melanoma cell lines A375, A875, M14 and SK28 and the normal human skin melanocyte line PIG1 were purchased from the American Type Tissue Culture Collection (ATCC) (Manassas, VA, United States). A375, A875, M14 and SK28 cells were cultured in DMEM (Invitrogen, New York, United States), and the PIG1 cell line was cultured in RPMI 1640 media (Invitrogen, New York, United States) supplemented with 10% certified heat-inactivated fetal bovine serum (FBS; Gibco) and 100 U/mL penicillin/streptomycin (Gibco, Langley, United States) and incubated at 37°C in a humidified incubator containing 5% CO_2_ (Thermo Fisher Scientific, Kalamazoo, MI). For *in vitro* knockdown of MCM2 in A375, A875 and SK28 cells, human MCM2 and control nonsense siRNAs were transfected into A375, A875 and SK28 cells. The siRNAs targeting MCM2 were purchased from RiboBio (Guangzhou, China). Transfection of siRNAs was performed according to the manufacturer’s protocol. Briefly, cells in the exponential phase of growth were plated in six-well tissue culture plates and then transfected with siRNAs using lipofectamine 3,000 (Invitrogen, New York, United States) reagent according to the manufacturer’s protocol.

### Western Blotting

Total protein was lysed on ice with 1% RIPA lysis buffer (Yeasen, Shanghai, China) for 30 min to prepare the cell suspension; the sample was centrifuged at 12,000 rpm for 15 min at 4°C, and the protein concentration was quantified with a BCA kit (Yeasen, Shanghai, China). Proteins were separated by 10% SDS–PAGE and transblotted to PVDF membranes (Millipore, Billerica, MA, United States). After being blocked in 5% nonfat milk for 1 h at room temperature, the membranes were incubated with primary antibodies (MCM2: 1:1,000 dilution, Cat No. YM0069, ImmunoWay, United States; MCM3: 1:1,000 dilution, Cat No. YT5217, ImmunoWay, United States; MCM4: 1:1,000 dilution, Cat No. YT2681, ImmunoWay, United States; MCM5: 1:1,000 dilution, Cat No. YT0812, ImmunoWay, United States; MCM6: 1:1,000 dilution, Cat No. YT5454, ImmunoWay, United States; MCM7: 1:1,000 dilution, Cat No. YT5371, ImmunoWay, United States; Actin: 1:5,000 dilution, Cat No. 23660-1-AP, Proteintech, China; Akt: 1:1,000 dilution, Cat No. 4685, CST, United States; pAkt:1:1,000 dilution, Cat No.13038, CST, United States) at 4°C with gentle shaking overnight. After the membranes were washed three times for 20 min each, secondary antibodies (goat anti-rabbit immunoglobulin G: 1:50,000 dilution, Cat No.111-035-003, Jackson ImmunoResearch, United States and goat anti-mouse immunoglobulin G 1:50,000 dilution, Cat No. 115-0050205, Jackson ImmunoResearch, United States) were added to the membranes and incubated at room temperature for 1 h. The blots were visualized with ECL reagent (Yeasen, Shanghai, China) according to the manufacturer’s protocol.

### CCK-8 Assay

After transfection with siRNA for 48 h, the cells were transferred to 96-well plates (100 µl cell suspension per well) at a density of 3,000 cells/well in triplicate for each group and incubated in a humidified incubator (Thermo Fisher). CCK-8 reagent (Beyotime, shanghai, China) was added to each well, and the cells were incubated for two additional hours. An iD3 microplate reader was used to measure the absorbance [optical density (OD) value] at 450 nm. The OD value was measured at 0, 24, 48, 72 and 96 h.

### Statistical Analysis

Data are presented as the mean ± SD using SPSS 19.0 (SPSS, Inc., Chicago, IL, USA). *p* values <0.05 were considered statistically significant. The differences between groups were statistically evaluated by Student’s *t* test. A two-tailed *p* < 0.05 was considered to indicate significance in all tests.

## Results

### Expression Levels of MCM2 Across Cancers

**FIGURE 2:**
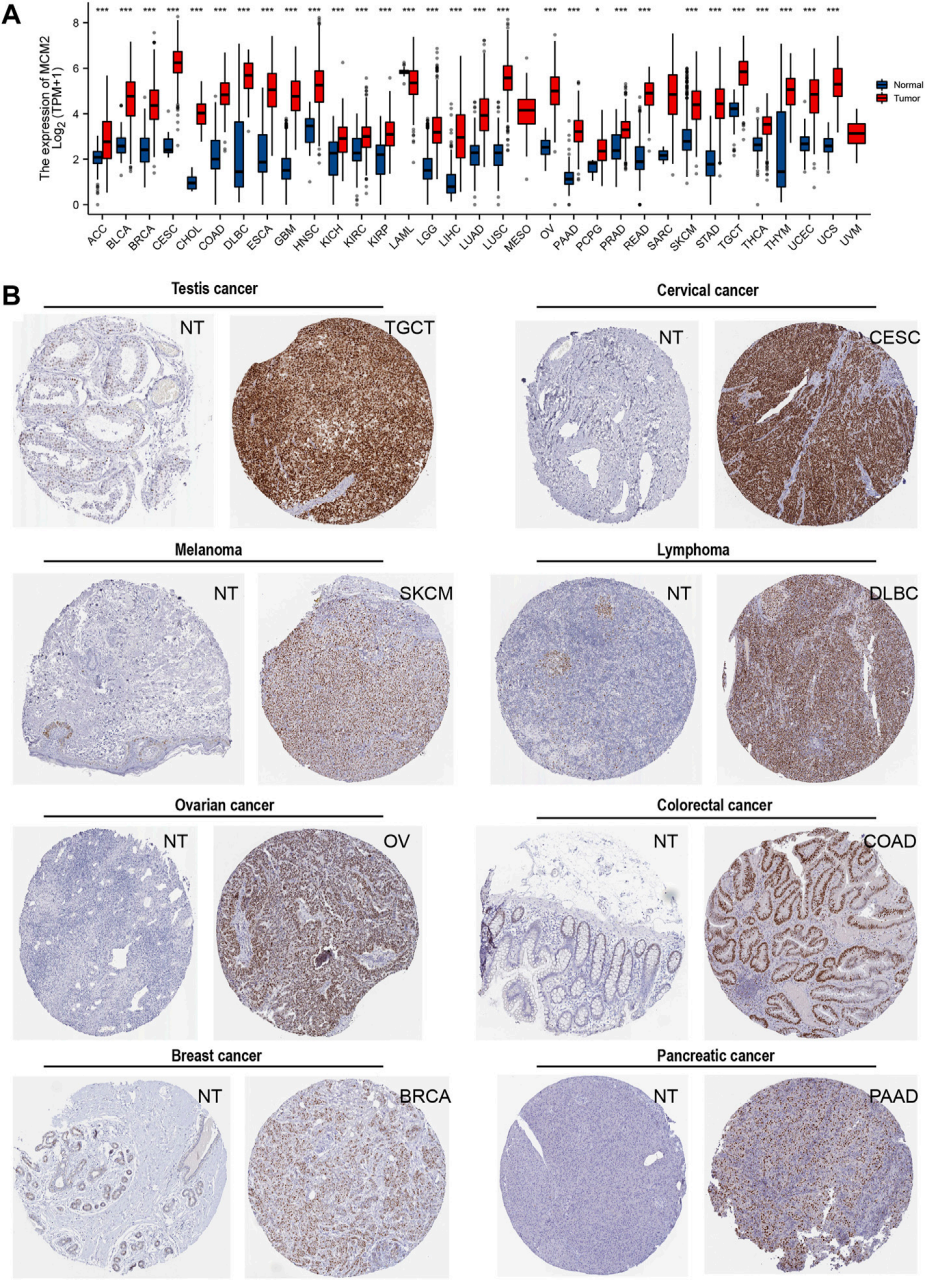
The expression of MCM2 in human cancers. **(A)** mRNA expression of MCM2 across cancers from TCGA in adrenocortical carcinoma (ACC), bladder urothelial carcinoma (BLCA), breast invasive carcinoma (BRCA), cervical squamous cell carcinoma and endocervical adenocarcinoma (CESC), cholangiocarcinoma (CHOL), colon adenocarcinoma (COAD), lymphoid neoplasm diffuse large B-cell lymphoma (DLBC), esophageal carcinoma (ESCA), glioblastoma multiforme (GBM), head and neck squamous cell carcinoma (HNSC), kidney chromophobe (KICH), kidney renal clear cell carcinoma (KIRC), kidney renal papillary cell carcinoma (KIRP), acute myeloid leukemia (LAML), brain lower‐grade glioma (LGG), liver hepatocellular carcinoma (LIHC), lung adenocarcinoma (LUAD), lung squamous cell carcinoma (LUSC), mesothelioma (MESO), ovarian serous cystadenocarcinoma (OV), pancreatic adenocarcinoma (PAAD), pheochromocytoma and paraganglioma (PCPG), prostate adenocarcinoma (PRAD), rectum adenocarcinoma (READ), sarcoma (SARC), skin cutaneous melanoma (SKCM), stomach adenocarcinoma (STAD), testicular germ cell tumors (TGCT), thyroid carcinoma (THCA), thymoma (THYM), uterine corpus endometrial carcinoma (UCEC), uterine carcinosarcoma (UCS), uveal melanoma (UVM). **(B)** The protein expression of MCM2 in cancers from the HPA database (IHC).

**FIGURE 3:**
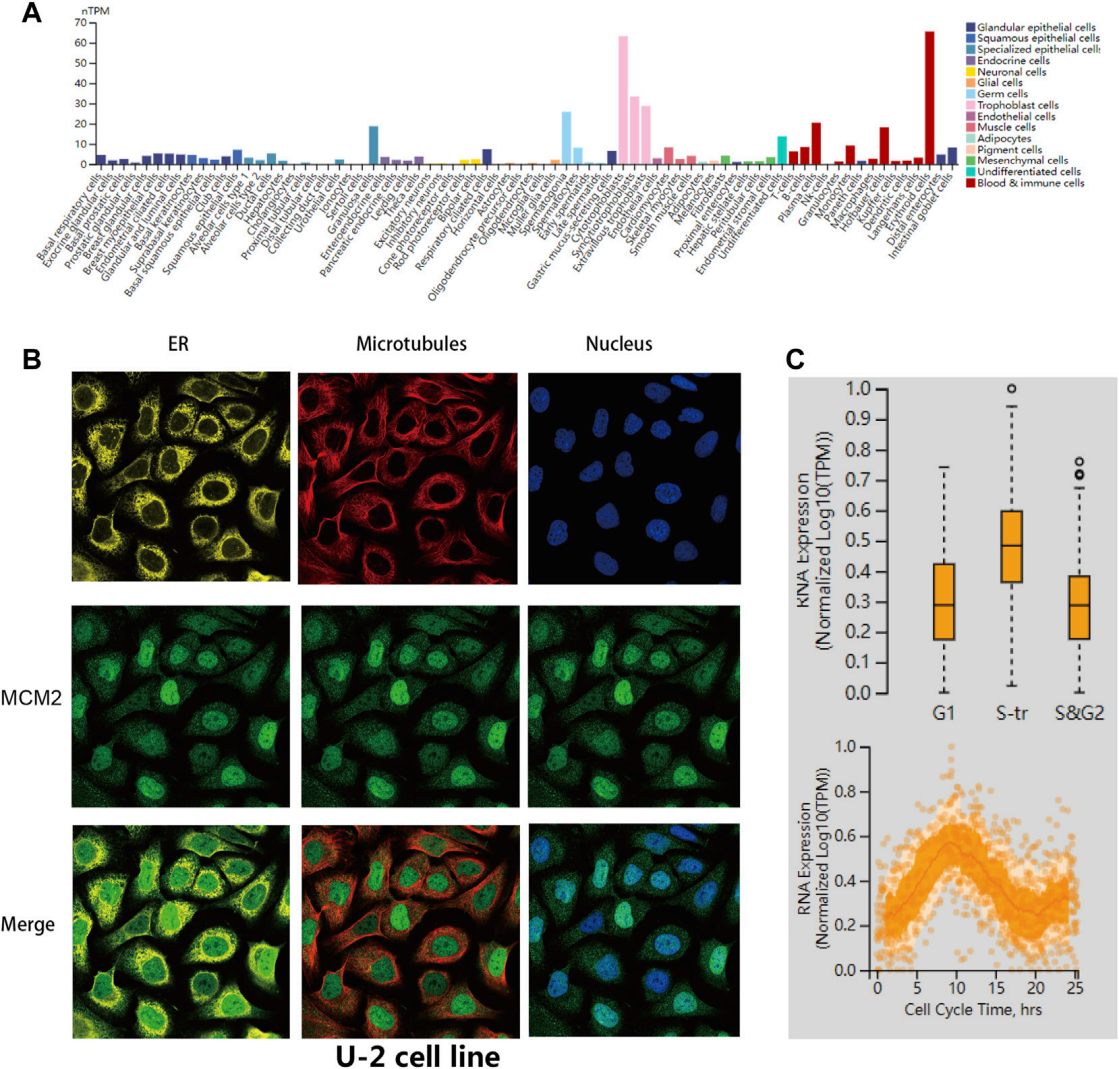
MCM2 variants, localization and single-cell variations. **(A)** MCM2 expression in normal cell lines from the HPA database. **(B)** Subcellular distribution of MCM2 within the endoplasmic reticulum (ER), nucleus and microtubules of the U-2 cells lines, according to the HPA database. **(C)** Plots of single-cell RNA-sequencing data from the FUCCI U-2 cell line showing the correlation between MCM2 RNA expression and cell cycle progression.

The transcription levels of MCM2 in different cancers and corresponding adjacent normal tissues were analyzed with R studio, combining data from TCGA and GTEx (Figure 2A). The mRNA expression level of MCM2 was markedly upregulated in patients with 33 cancers (*p* < 0.05), including ACC, BLCA, BRCA, CESC, CHOL, COAD, DLBC, ESCA, GBM, HNSC, KICH, KIRC, KIRP, LAML, LGG, LIHC, LUAD, LUSC, OV, PAAD, PRAD, READ, SKCM, STAD, TGCA, THCA, THYM, UCEC, and UCS (Figure 2A). In addition, we analyzed IHC data from the HPA database to investigate the expression of MCM2 at the protein level. The staining and intensity data revealed that the expression levels of MCM2 were strikingly higher and stronger in TGCT, CESC, SKCM, DLBC, OV, COAD, BRCA and PAAD tissues compared to normal tissues (Figure 2B). In the cell lines of normal tissues, MCM2 expression was low across most cell lines, except in a few cell lines, such as granulosa cells, spermatocytes, cytotrophoblasts, syncytiotrophoblasts, extravillous trophoblasts, undifferentiated cells, plasma cells, Hofbauer cells and erythroid cells (Figure 3A). Taken together, these results indicate that MCM2 is widely overexpressed across cancers based on different databases, and the protein levels were consistent with their mRNA expression levels in BRCA, CESC, COAD, HNSC, LIHC, LUSC, OV, PAAD, SKCM, STAD, TGCT, and UCEC.

Furthermore, we verified the intracellular localization of MCM2 within the endoplasmic reticulum (ER), microtubules and nucleus of U-2 cell lines. We observed that MCM2 was mainly located in the nucleus, which is consistent with previous studies, while there was no overlap with the ER and microtubules (Figure 3B). Single-cell RNA-sequencing data from the Fluorescent Ubiquitination-based Cell Cycle Indicator (FUCCI) showed that MCM2 RNA expression was significantly correlated with cell cycle progression through the G1, S and G2 phase (Figure 3C).

### Correlations Between MCM2 and Molecular or Immune Cancer Subtypes

**FIGURE 4:**
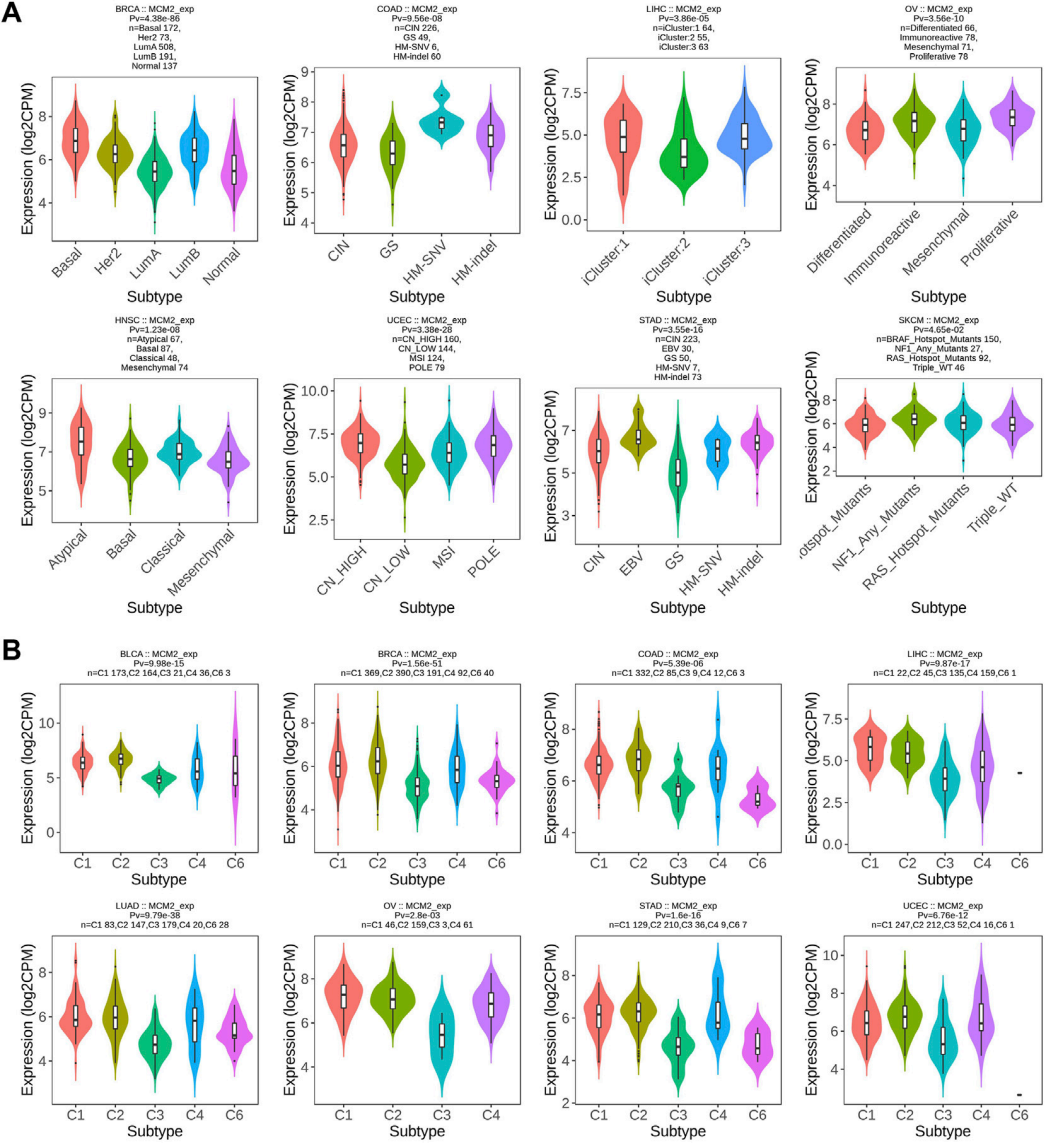
Correlations between MCM2 expression and **(A)** molecular subtypes and **(B)** immune subtypes across TCGA cancers. CIN, chromosomal instability; GS, genomically stable; POLE, Polymerase ε; EBV, Epstein-Barr virus.

Molecular subtypes were observed to be related to MCM2 expression in eight cancers: BRCA, COAD, HNSC, LIHC, OV, SKCM, STAD and UCEC. MCM2 was expressed at higher levels in the basal molecular subtype than the other molecular subtypes of BRCA, slightly higher in the HM-SNV and HM-indel molecular subtypes of COAD, highly expressed in the iCluster:1 and iCluster:3 molecular subtypes of LIHC and expressed at the highest level in the proliferative cell molecular subtypes of OV. For HNSC, MCM2 was expressed at the highest level in the atypical molecular subtype. For UCECs, MCM2 expression was slightly higher in CN_HIGH and POLE cells. MCM2 was upregulated in the EBV molecular subtype of STAD and the NF1_Any_Mutants molecular subtype of SKCM (Figure 4A). In addition, immunotherapy was also effective in treating various malignancies. Therefore, immune subtypes of certain cancers are critical characteristics of the immunotherapy response and are predictive of outcome. According to previous studies, immune subtypes include C1: wound healing, C2: IFN-gamma dominant, C3: inflammatory, C4: lymphocyte depleted, C5: immunologically quiet, and C6: TGF-b dominant (Thorsson et al., 2019). MCM2 expression was significantly correlated with the immune subtypes of eight cancers, including BLCA, BRCA, COAD, LIHC,, LUAD, OV, STAD and UCEC (Figure 4B).

### Genetic Alteration Analysis of MCM2

**FIGURE 5:**
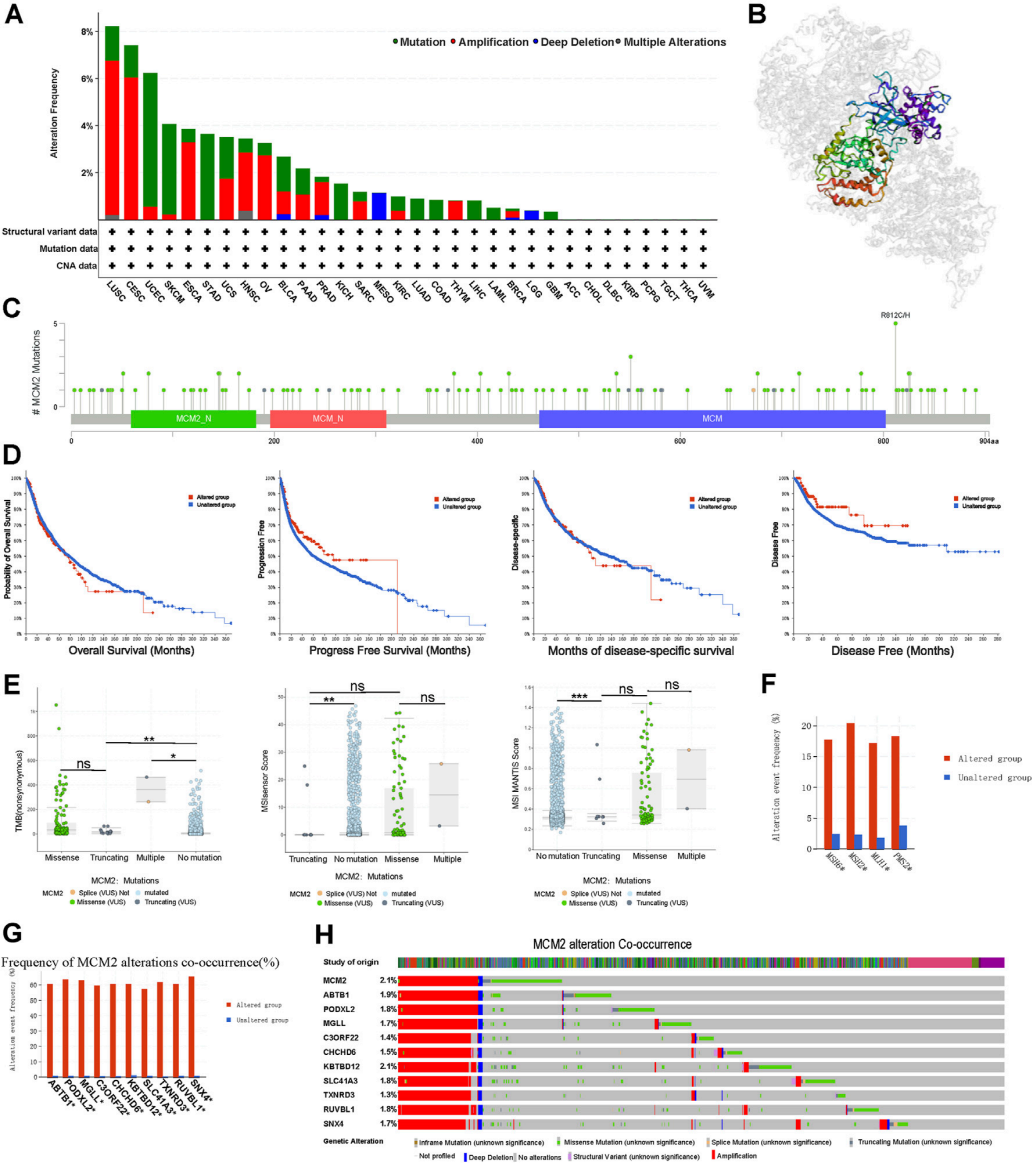
MCM2 gene alterations in various cancers. **(A)** MCM2 gene mutation type analysis in various cancers by cBioPortal. **(B)** 3D protein structure of MCM2. Colored part means the binding region, while grey means the other part of MCM2. **(C)** The subtypes and distributions of MCM2 somatic mutations. *X*-axis, amino acids site; *Y*-axis, number of MCM2 mutations; green dot, missense mutations; grey dot, truncating mutations. **(D)** The correlation between mutation status and patient prognosis for all TCGA cancers, including overall survival, progression-free survival, disease-specific survival and disease-free survival, analyzed using the cBioPortal tool. Red square indicated the MCM2 alteration group, Blue square indicated non-alteration group. **(E)** TMB and MSIsensor scores and MSI MANTIS scores in MCM2 nonmutant cancer and different subtypes of MCM2 mutant cancer (including truncating, missense and multiple). Each dot represents one patient. The black line represents the median and the interquartile ranges. *X* axes indicated different mutation type of MCM2. *Y* axes indicated scores of cases. **(F)** The mutant frequencies of MSH6, MSH2, MLH1 and PMS2 in the MCM2 mutant and nonmutant groups. **(G)** Bar plot showing the frequencies of the ABTB1, PODXL2, MGLL, C3ORF22, CHCHD6, KBTBD12, SLC41A3, TXNRD3, RUVBL1 and SNX4 alterations co-occurring with MCM2 alterations. **(H)** Waterfall plot showing the cooccurrence patterns of MCM2 alterations with the genetic alterations of ABTB1, PODXL2, MGLL, C3ORF22, CHCHD6, KBTBD12, SLC41A3, TXNRD3, RUVBL1 and SNX4. Color indicated the different mutation type of the 11 genes in cases.

The prevalence of MCM2 somatic mutations was analyzed to determine the MCM2 mutation rate in clinical samples across 32 cancers. MCM2 mutated in many cancers, except ACC, CHOL, DLBC, KIRP, PCPG, TGCT, THCA, and UVM (Figure 5A). The most frequent mutation types were amplification and mutation. In addition, DNA alteration can result in protein primary structure changes or amino acid changes. Figure 5B showed the 3D structure of MCM2. As shown in Figure 5C, there were multiple dispersed mutation sites in the amino acids of MCM2, of which the most common mutation type was missense mutation. Moreover, the relationship between MCM2 genetic mutations and the clinical survival prognosis among patients was analyzed across cancers. An improved probability of OS and progression-free and disease-specific survival was observed in the MCM2-unaltered group compared to patients with MCM2 alterations but not in the disease-free group (Figure 5D). We then conducted gene correlation analysis and determined the top 10 other genes that were near-perfectly correlated with MCM2, including ABTB1, PODXL2, MGLL, C3ORF22, CHCHD6, KBTBD12, SLC41A3, TXNRD3, RUVBL1 and SNX4. The frequency and pattern of genetic alterations in MCM2 co-occurred with these same genes, indicating that these genes are associated with MCM2 in the promoting cancer development (Figures 5G,H).

The TMB referred to the number of somatic mutations in the tumor genome except for germline mutations (Rizvi et al., 2015). In the TCGA cohort, higher TMB was correlated with different types of MCM2 mutations across cancers, which were significantly different among cancers with no mutation, truncating mutants, missense mutants or multiple mutations. In general, TMB was higher in patients with MCM2 mutant cancers (median: 21.23; interquartile range: 9.72–86.23) than MCM2 nonmutant cancers (0.63, 0.87–4.37; *p* < 0.05). Moreover, the TMB was significantly different among cancers with multiple MCM2 mutations (364, 313.9–414.08), truncating MCM2 mutations (17.5, 8.84–30.89) and missense MCM2 mutations (35.63, 10.65–90.36; Figure 5E). The level of the microsatellite instability sensor (MSIsensor) was used to evaluate the MSI status of the tumor. Patients with MCM2 mutant cancers had higher MSIsensor scores (3.26; 0.045–17.17) than those with MCM2 nonmutant cancers (0.05, 0–0.31; *p* < 0.05; Figure 5E). The MSIsensor score was much higher in cancers with multiple MCM2 mutations (14.53; 8.90–20.17) than those with missense MCM2 mutations (0.04; 0.07–17.06) and truncating MCM2 mutations (0.03; 0.01–0.05). The correlation between MSIsensor scores and MCM2 mutations were further comfirmed by examining the MSI MANTIS score, which predicts a patient’s MSI status (Bonneville et al., 2017). MSI status is normally classified as microsatellite instability high (MSI-H), microsatellite instability low (MSI-L) or microsatellite stable (MSS). The MSI MANTIS score had a positive correlation with the probability of MSI-H status (Lu et al., 2021). Similarly, the MSI MANTIS score was higher in cancers with multiple MCM2 mutations (0.32; 0.30–0.74) than no MCM2 mutations (0.30; 0.29–0.33, *p* < 0.05) (Figure 5E). Cancer cells lack DNA mismatch repair machinery, including genes such as MSH6, MLH1, MSH2 and PMS2, which leads to replication errors persist in tumor cells (Niu et al., 2014). MSH2, MSH6, MLH1, and PMS2 are reported as vital proteins for the mismatch repair (MMR) process. Thus, co-occurrence analysis was conducted between these four MMR genes and MCM2 alterations. MCM2 mutation group had a significantly higher mutant frequency of these four MMR genes comparing to patients without MCM2 mutations (Figure 5F).

To understand MCM2 mutations across cancers, different mutation types and single nucleotide variants (SNVs) were identified. The highest frequency of MCM2 mutation was missense substitution in BRCA (26.19%), CESC (80%), LIHC (37.5%), LUAC (51.52), OV (47.37%), SKCM (55.79%), and PAAC (37.5%) but not PRCA (5%). Synonymous substitution was also common in some cancers, such as LUAC (30.3%) and SKCM (35.79%) (Supplementary Figure S1). However, nonsense substitutions and other mutations were rare and only found in a few out of the total samples. SNV data showed that the primary class type was G > A in BRCA (41.18%), CESC (40%), LIHC (19.05%) and OV (25%), while the most common SNVs in LUAC were C > T (20%) and G > T (21.82%), C > T (65%) in SKCM, C > T (50%) in PAAC, and A > G (25%), C > T (25%), G > A (25%) and G > T (25%) in PRCA (Supplementary Figure S1).

**FIGURE 6:**
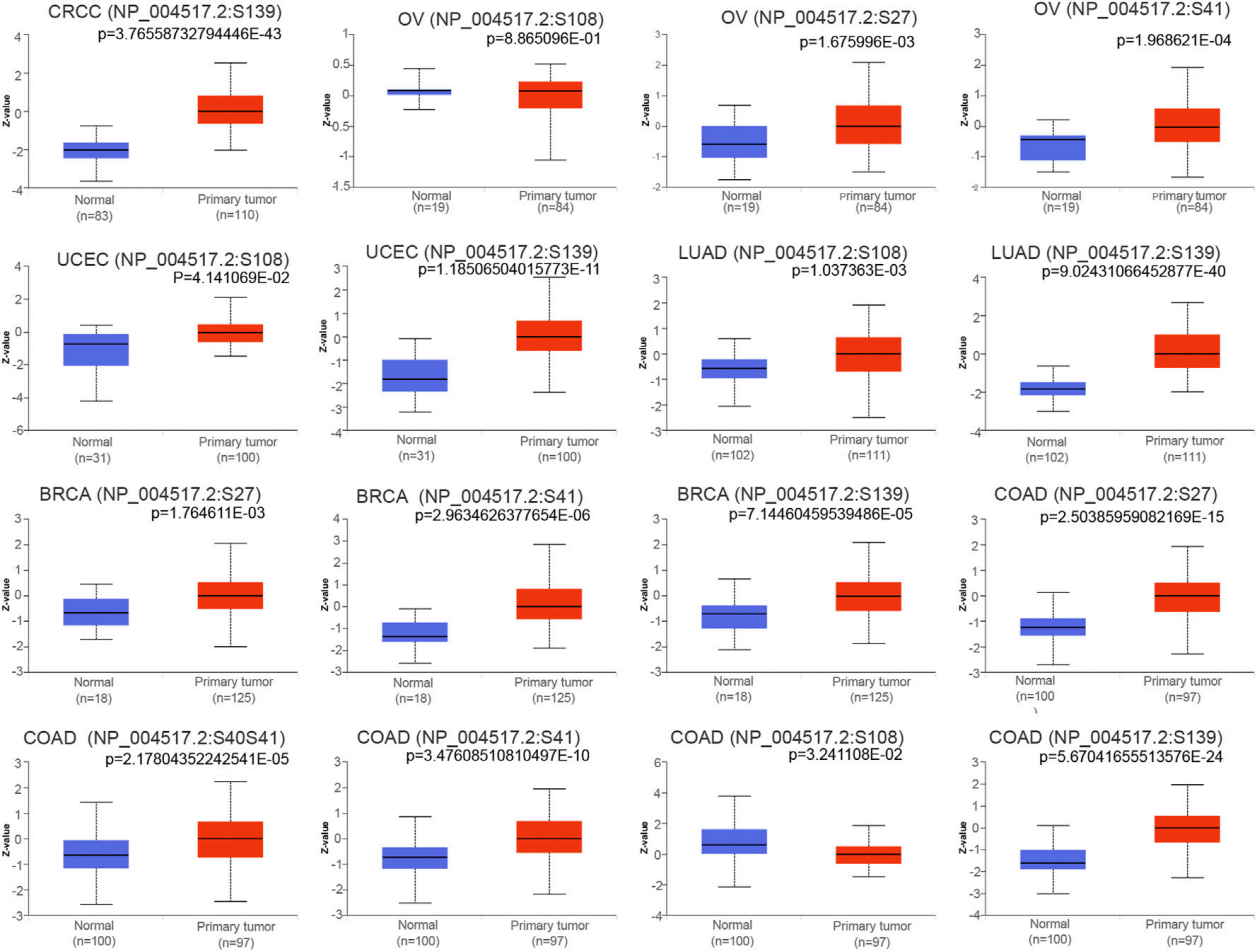
Boxplots showing differential MCM2 phosphorylation levels (beta values) between tumors and adjacent normal tissues across TCGA database.

Protein phosphorylation is an important posttranslational modification process that plays a key role in the regulation of protein molecule activity, second messenger transmission and enzyme cascade reactions (Kataya et al., 2019). We determined whether there were any changes in the phosphorylation level of MCM2 across cancers. Higher phosphorylation levels of loci S27 and S41 of MCM2 protein were exhibited in OV tissue (all *p* < 0.05), while there was no significant difference in locus S108 of MCM2 (*p* = 0.88) (Figure 6). For BRCA, phosphorylation of S27, S41 and S139 in MCM2 was significantly higher in tumor tissue (all *p* < 0.05). Multiple MCM2 phosphorylation sites significantly differed between COAD tissues and normal tissues, such as S27 (*p* = 2.50E-15), S40S41 (*p* = 2.17E-05), S41 (*p* = 3.47E-10), S108 (*p* = 5.67E-24) and S139 (*p* = 5.67E-24). The MCM2 phosphorylation locus of S139 (*p* = 3.76E-43) exhibited a higher phosphorylation level in CRCC tissues. The S108 (*p* = 4.14E-02, *p* = 1.03E-03) and S139 (*p* = 1.18E-11, *p* = 9.02E-40) loci were promising functional sites that were significantly differentially expressed between UCEC and LUAD cancer tissues and normal tissues (Figure 6). In summary, these findings illustrated the different phosphorylation sites of MCM2 and highlight potential loci for further molecular assays in a subset of cancers.

### Functional and Pathway Enrichment Analysis of MCM2 Across Cancers

**FIGURE 7:**
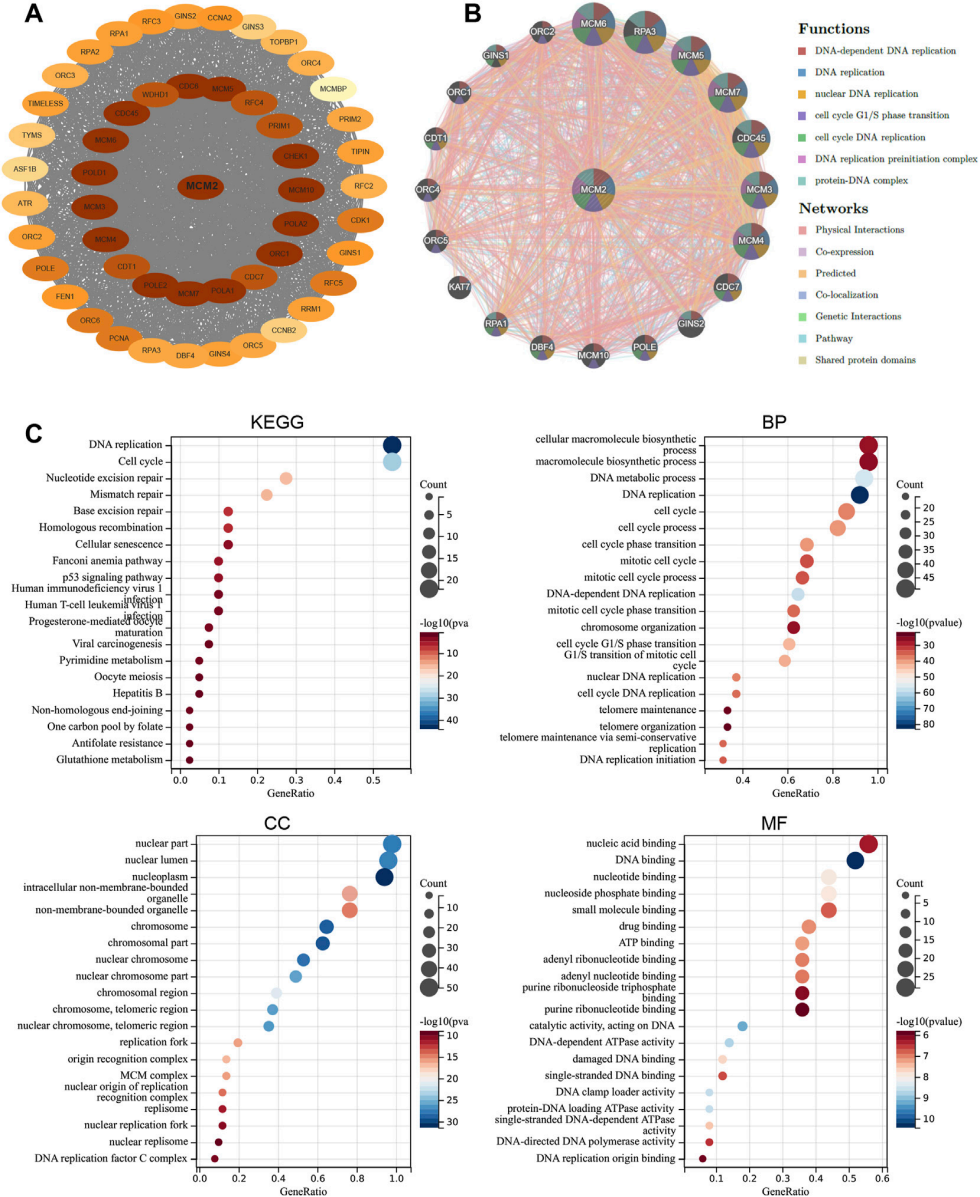
Functional enrichment and co-expression network of MCM2 at the gene and protein levels. **(A)** PPI network. The related proteins of MCM2. The color depth revealed the correlation between MCM2 and other proteins, which means that the darker the color, the closer the relationship. **(B)** GGI network. **(C)** KEGG and GO analyses of 50 targeted binding proteins of MCM2 in patients with cancers.

To order to explore the function of MCM2 in pan-cancers, a PPI network was used to reveal the relationship between MCM2 and the functional-related proteins. 20 highly related genes and 31 less highly related proteins were present in the network (Figure 7A). These related proteins are mostly involved in DNA replication and cell cycle progression, especially MCM3-7 and MCM10, which are members of the MCM family. Subsequently, a GGI network was used to reveal the relationship between MCM2 and its neighboring genes (Figure 7B). There were 20 representative genes that were strongly related to MCM2. These neighboring genes are mainly involved in DNA replication, cell cycle transition and DNA-related complexes, and the networks are mainly based on physical interactions, co-expression, prediction, colocalization, genetic interactions, pathways and shared protein domains. The KEGG analysis showed that MCM2 was enriched in pathways like cell cycle and DNA replication. GO enrichment analysis indicated that 20 biological processes were enriched (with adjusted *p* < 0.05). MCM2 was mainly enriched in cellular macromolecule biosynthesis, DNA metabolism, DNA replication and cell cycle processes. MCM2 participates in various cellular components located in the nucleus, nuclear lumen and nucleoplasm (Figure 7C). Molecular function analysis revealed that MCM2 and its related proteins were mainly enriched in nucleic, DNA and nucleotide binding.

### Analysis of the Clinical Value of MCM2 Across Cancers

**FIGURE 8:**
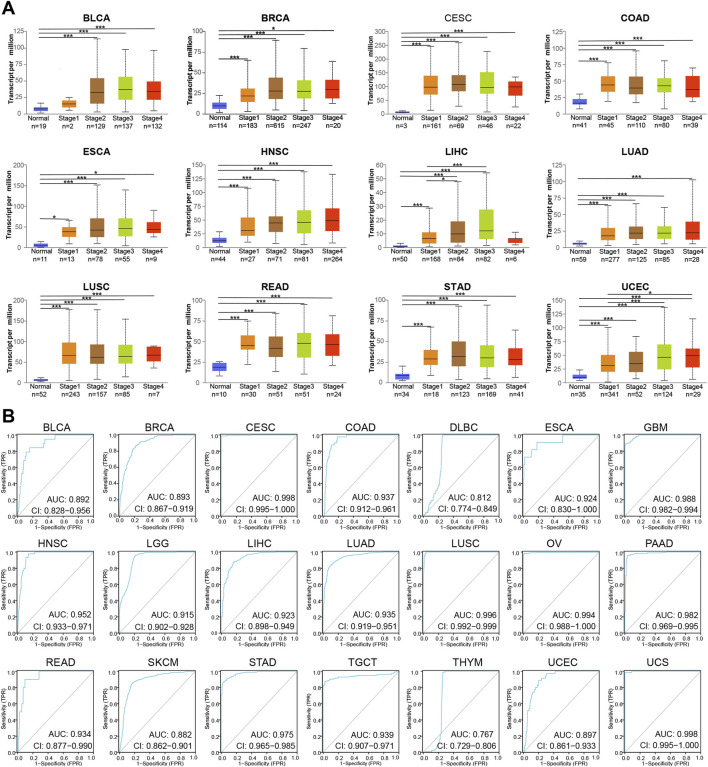
The prognostic value of MCM2 in various cancers. **(A)** The correlation between transcription levels of MCM2 and cancer stage in patients. **(B)** AUCs of MCM2 in predicting the prognosis of patients with various types of cancer.

To further reveal the clinical value of MCM2 across cancers, here evaluated four indicators. First, the correlation between the transcription levels of MCM2 and cancer stage was analyzed. The mRNA expression of MCM2 was significantly correlated with cancer stages of BLCA, BRCA, CESC, COAD, ESCA, HNSC, LIHC, LUAD, LUSC, READ, STAD and UCEC (*p* < 0.05) (Figure 8A). Most of these cancers had a significant difference in MCM2 transcription between normal tissue and different cancer stages. We also evaluated the predictive power of MCM2 and constructed ROC curves to assess the ability of MCM2 to predict the prognosis of patients with various cancers. Interestingly, the data showed that MCM2 alone had an AUC >0.7 in 21 cancers, and MCM2 exhibited the highest predictive value in the prognostic model of CESC (AUC 0.998, CI 0.995-1), LUSC (AUC 0.996, CI 0.992-0.999), OV (AUC 0.994, CI 0.988-1) and UCS (AUC 0.998, CI 0.995-1), but the lowest predictive value for the prognostic model of THYM (AUC 0.767, CI 0.729-0.806). In addition, in other 14 cancers, AUC value ranged from 0.812 to 0.952 (Figure 8B).

**FIGURE 9:**
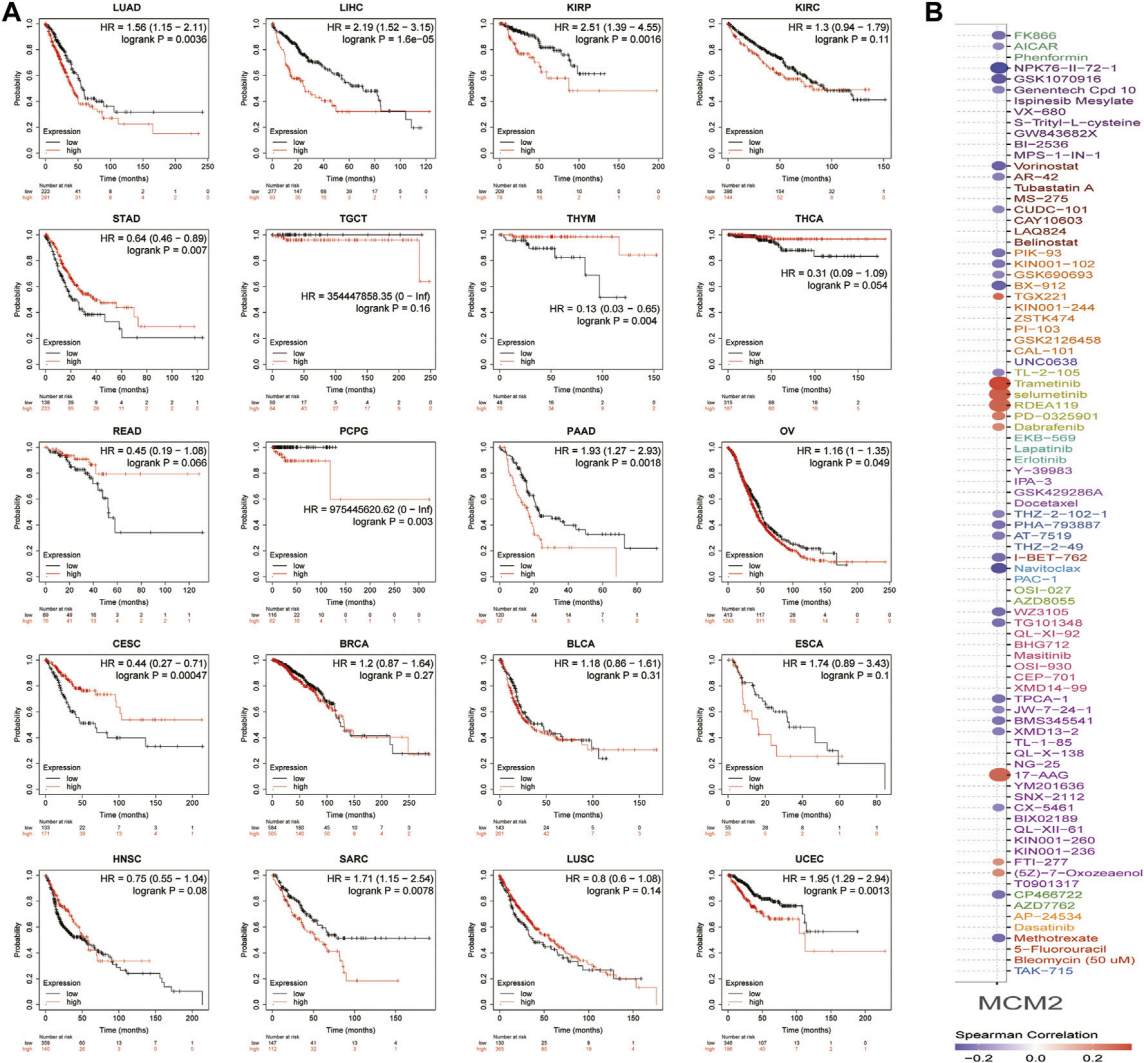
The clinical value of MCM2 in various cancers. **(A)** OS of patients with different expression levels of MCM2 in pan-cancer from the Kaplan–Meier Plotter database. **(B)** The relationship between MCM2 expression level and sensitivity to different drugs. The colors indicated the correlation between MCM2 expression and drug sensitivity. Red indicated positive relationship, while purple negative. The size of the spots indicated the significance of the correlation. The bigger the size, the more significant correlation.

Then, the prognostic value of MCM2 expression in patients with various cancers was analyzed. The data showed that higher expression of MCM2 was significantly associated with worse OS rate in KIRP (*p* = 0.0016, HR = 2.51), LIHC (*p* = 1.6e-05, HR = 2.19), LUAC (*p* = 0.00367, HR = 1.56), PDAC (*p* = 0.0018, HR = 1.93), PCPG (*p* = 0.003, HR = 975445620.62), SARC (*p* = 0.0078 HR = 1.71), UCEC (*p* = 0.0013, HR = 1.95) and OV (*p* = 0.049, HR = 1.16). Therefore, higher levels of MCM2 predicted worse OS across cancers (Figure 9A). Additionally, we analyzed the sensitivity of MCM2 to anticancer drugs in various cancers by using genomics of drug sensitivity, which indicated that MCM2 could serve as potential biomarkers for drug screening and affect clinical responses to treatment. The red dot indicated the positive correlation between gene expression and the resistance to drugs. The purple dot indicated negative correlation. Higher MCM2 level was resistant to nine drugs or small molecules, such as Trametinib, and sensitive to 27 drugs, such as NPK76-II-72-1, suggesting that MCM2 may be a potential biomarker for drug screening across cancers (Figure 9B).

### The Relationship Between MCM2 and Immune Infiltration

**FIGURE 10:**
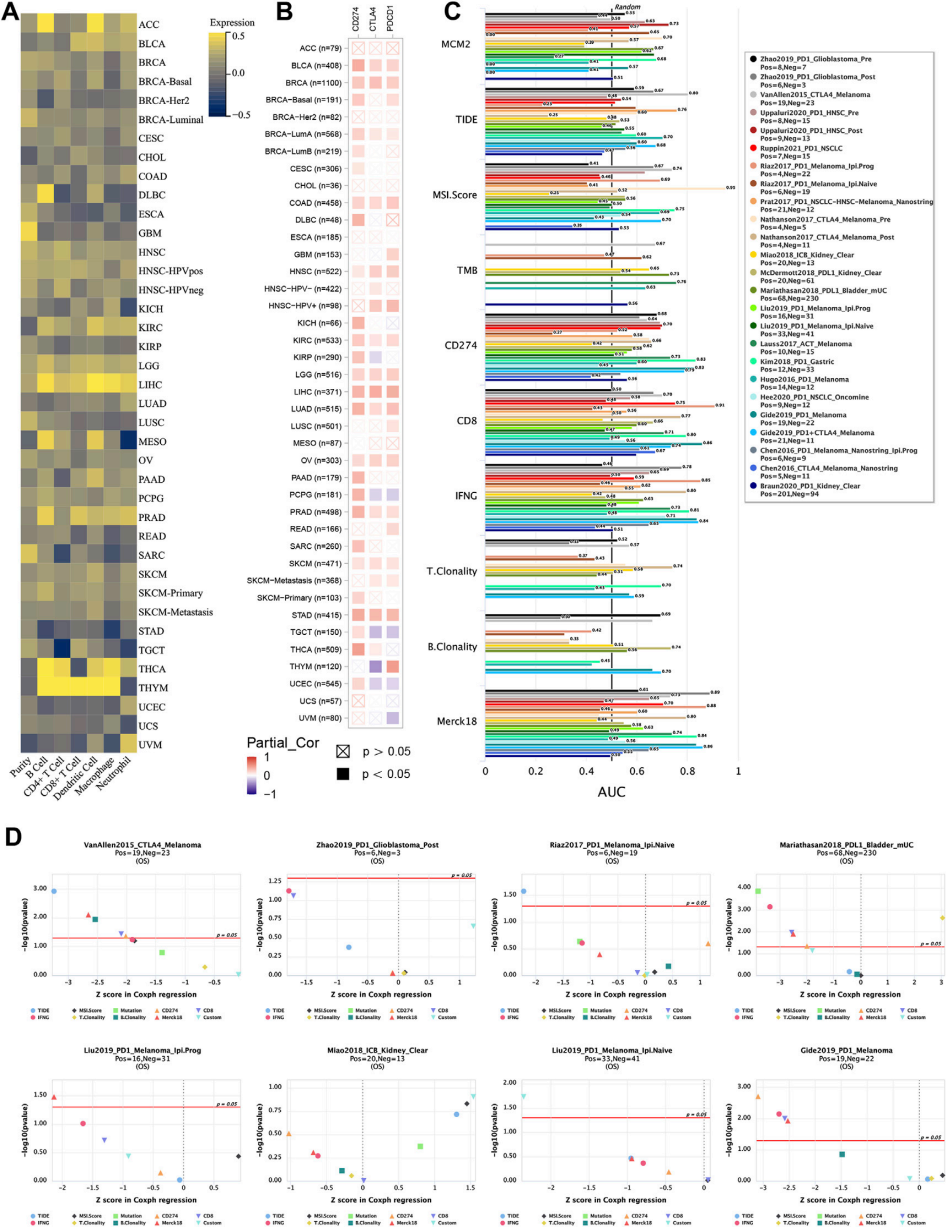
MCM2 expression and immune infiltration in various TCGA cancers. **(A)** Correlations between MCM2 expression and cancer purity and infiltrating levels of B cells, CD8^+^ T cells, CD4^+^ T cells, macrophages, neutrophils and dendritic cells. **(B)** Correlations between MCM2 expression and CD274, CTLA4 and PDCD1. **(C)** Bar plot showing the biomarker relevance of MCM2 compared to that of standardized cancer immune evasion biomarkers in immune checkpoint blockade (ICB) subcohorts. The AUC was applied to evaluate the predictive performance of the test biomarkers on the ICB response status. **(D)** Comparison between MCM2 and other published biomarkers based on their predictive power of response outcome and OS. Color indicated the different immune biomarkers.

Tumor immunotherapy has been recognized as a promising treatment that is influenced by the immune microenvironment. The correlation was evaluated between MCM2 expression and the infiltration of six immune cell types (B cells, CD4^+^ T cells, CD8^+^ T cells, dendritic cells, macrophages and neutrophils) and the purity in 32 TCGA cancers and subtypes. However, MCM2 expression positively correlated with all six immune cell infiltrates in only four cancers (HNSC, LGG, LIHC, PRAD). LIHC had a strong positive correlation (*r* = 0.21–0.49, all *p* < 0.05), while HNSC (*r* = 0.11–0.27, all *p* < 0.05), LGG (*r* = 0.19–0.31, all *p* < 0.05) and PRAD (*r* = 0.08–0.39, all *p* < 0.05) had weak positive correlations. THYM exhibited strong positive correlation between MCM2 expression levels and infiltration of all immune cells except neutrophils (*r* = 0.49–0.78, all *p* < 0.05), and THCA also had strong positive correlations between MCM2 expression levels and all immune cells except CD8^+^ T cells (*r* = 0.27–0.59, all *p* < 0.05). Other cancers had a negative (r < 0, *p* < 0.05) or no significant correlation (*p* > 0.05) (Figure 10A, Table 1, Supplementary Table S1).

**TABLE 1:** Correlation between cancers and immune cells.

Cancer	Purity	B Cell	CD4+ T Cell	CD8+ T Cell	Dendritic Cell	Macrophage	Neutrophil
ACC	0.154363	0.487521	0.060736	0.106755	0.476625	0.194369	0.309513
BLCA	−0.00829	0.088769	−0.0119	0.309518	0.315922	0.215788	0.249852
BRCA	0.228238	0.209145	0.133949	0.040109	0.179677	−0.05178	0.166373
BRCA-Basal	0.167659	0.085952	0.209805	−0.0074	0.214877	−0.10749	0.163763
BRCA-Her2	0.130184	−0.17809	0.149821	−0.03897	−0.05654	−0.04012	0.01959
BRCA-Luminal	0.297655	0.108342	0.111153	0.061757	0.148506	0.047764	0.13438
CESC	0.10562	0.091595	0.168388	0.025929	0.092948	−0.04635	0.143756
CHOL	−0.14122	0.094296	−0.00743	0.183602	0.109438	−0.03742	−0.01957
COAD	0.075898	0.01581	0.157246	−0.07084	0.161759	0.082482	0.207756
DLBC	0.157095	0.45601	−0.19185	−0.06324	0.249392	−0.14416	−0.13091
ESCA	0.270084	0.084477	−0.05853	−0.12905	−0.14873	0.036748	−0.14689
GBM	0.440297	−0.06836	0.008541	−0.04321	0.117452	−0.05316	−0.00139
HNSC	0.285393	0.194093	0.278068	0.115654	0.18772	0.181709	0.153539
HNSC-HPVpos	0.242403	0.22024	0.24105	0.24233	0.185044	0.02801	0.204202
HNSC-HPVneg	0.235184	0.064927	0.26673	−0.0122	0.119643	0.174809	0.098209
KICH	0.12131	−0.01013	−0.08693	0.150837	0.028393	0.222444	−0.23335
KIRC	−0.11351	0.299108	0.216477	0.120881	0.350027	0.263522	0.293088
KIRP	0.147969	0.041163	−0.08342	0.04184	0.071084	−0.02773	−0.01652
LGG	0.193982	0.310322	0.186638	0.266151	0.288988	0.212821	0.242913
LIHC	0.210463	0.456545	0.3198	0.348215	0.495277	0.449565	0.386952
LUAD	0.029398	−0.05469	0.02177	0.102044	0.108016	0.016443	0.21262
LUSC	0.286594	0.112479	0.085738	−0.04708	−0.01499	−0.09772	−0.05264
MESO	−0.10467	0.413084	0.208204	−0.00094	0.381492	0.077756	−0.40014
OV	0.198174	0.089598	0.141878	−0.02488	0.13939	0.102475	0.089477
PAAD	0.032659	0.20051	−0.12945	0.178186	0.319341	0.10439	0.161055
PCPG	0.15631	0.235485	0.132266	0.112246	0.047651	0.186506	0.136389
PRAD	0.082282	0.397705	0.113488	0.34999	0.288252	0.29932	0.341808
READ	0.049615	0.101108	0.121547	−0.12063	0.132766	−0.13839	−0.03528
SARC	0.352436	0.134124	−0.25776	0.096446	−0.00603	−0.2041	−0.07665
SKCM	0.106201	0.170338	0.130414	0.14318	0.251778	0.024721	0.166764
SKCM-Primary	0.16522	0.272953	0.10773	0.183274	0.187037	0.051955	0.152465
SKCM-Metastasis	0.087746	0.103227	0.092092	0.079998	0.210345	−0.03548	0.107457
STAD	0.103538	−0.23812	−0.12403	−0.03651	−0.07019	−0.30445	−0.01558
TGCT	0.215745	0.051931	−0.42888	0.234552	0.084423	−0.09675	−0.27425
THCA	−0.06888	0.597688	0.421514	−0.18809	0.36814	0.450128	0.279334
THYM	−0.09279	0.782967	0.498549	0.531257	0.653071	0.555405	−0.19327
UCEC	0.060696	−0.12919	−0.06004	−0.08894	−0.06579	−0.14001	0.2302
UCS	0.08006	0.043247	0.05325	−0.04795	−0.06644	0.089283	−0.18707
UVM	−0.19728	−0.0989	−0.03878	−0.1731	0.140775	−0.20733	0.348397

Recently, several genes that have been explored as immune checkpoints, called immune checkpoint genes (ICGs), are linked with cancer immune infiltration, immunotherapy responsibility and survival rate. Here, we evaluated the correlation between three classic ICGs, CD274, CTLA4 and PDCD1, and MCM2 expression in 40 TCGA cancers. MCM2 expression was strongly correlated with all three ICGs in BLCA, BRCA, CLAD, HNSC, KIRC, LGG, LIHC, LUAD, OV, PRAD, SKCM and STAD (all *p* < 0.5), while no significant correlation was showed in ACC, BRCA-HER2, CHOL or ESCA. Other cancers had a weak correlation between MCM2 expression and 1 or 2 ICGs (Figure 10B).

To further evaluate the biomarker potential of MCM2, we compared it with standardized biomarkers and examined their predictive power for response outcomes of ICB subcohorts. Interestingly, the data showed that MCM2 alone had an AUC >0.5 in 13 of the 25 ICB subcohorts. MCM2 exhibited a higher predictive value than TMB, T. Clonality, and B. Clonality, which had AUC values >0.5 in 8, 7, and 7 ICB subcohorts, respectively. However, MCM2 was comparable to the MSI score (AUC >0.5 in 14 ICB subcohorts), TIDE (AUC >0.5 in 18 ICB subcohorts), IFNG (AUC >0.5 in 17 ICB subcohorts) and Merck18 (AUC >0.5 in 18 ICB subcohorts) but lower than CD274 (AUC >0.5 in 21 ICB subcohorts) and CD8 (AUC >0.5 in 20 ICB subcohorts) (Figure 10C). We next analyzed the ability of MCM2 to predict the patient OS rate by comparing the Z score from Cox-PH regression with other published biomarkers (Jiang et al., 2018). Our results suggested that MCM2 has a good ability to predict patient OS across cancers (Figure 10D).

### MCM2 in SKCM

**FIGURE 11:**
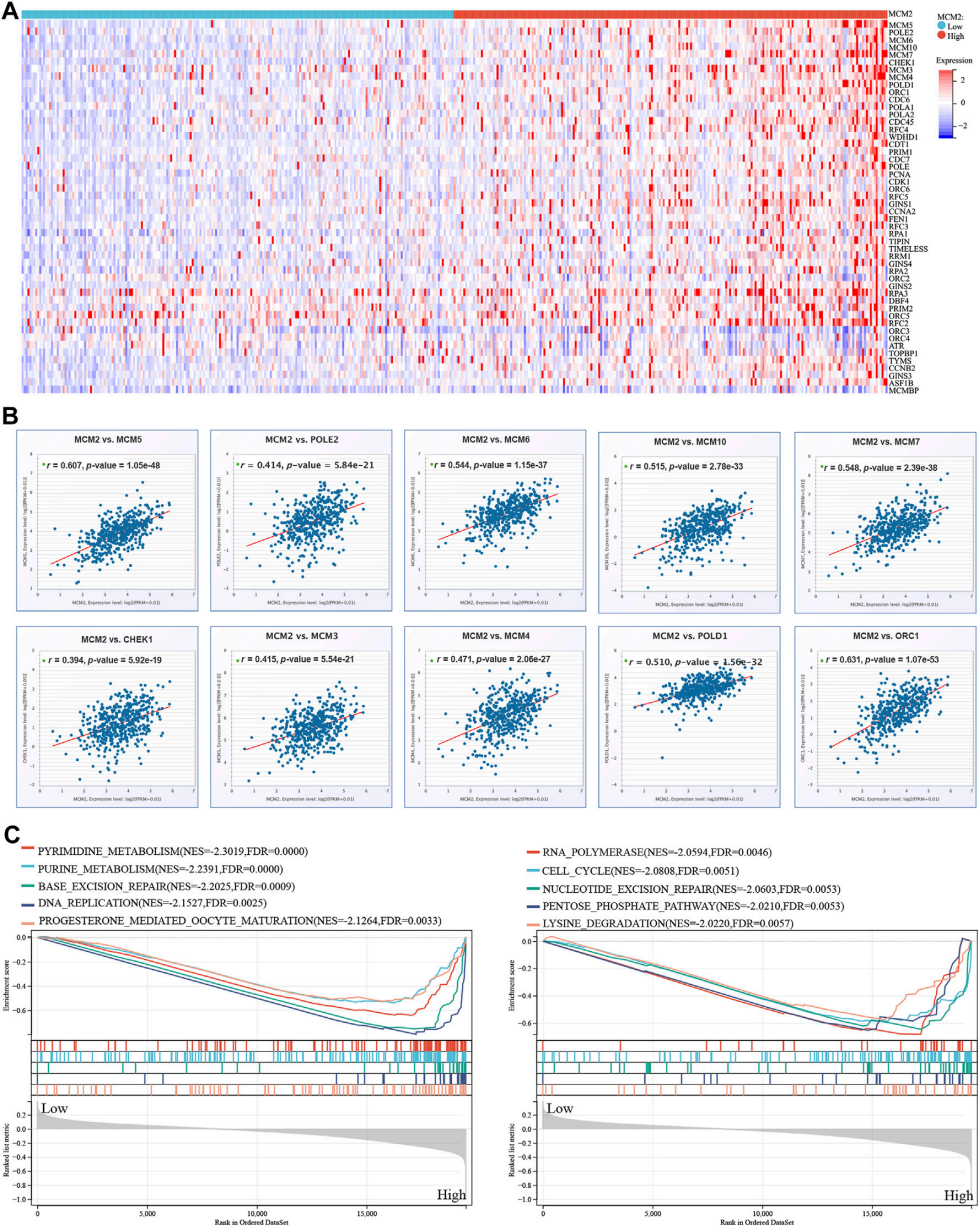
MCM2 in SKCM. **(A)** The top 50 genes with positive co-expression of MCM2 in the TCGA database of SKCM according to the heatmap. **(B)** The top 10 genes with a strong positive correlation with MCM2 in SKCM. **(C)** GSEA showing that MCM2 expression was associated with 10 pathways in TCGA SKCM samples.

**FIGURE 12:**
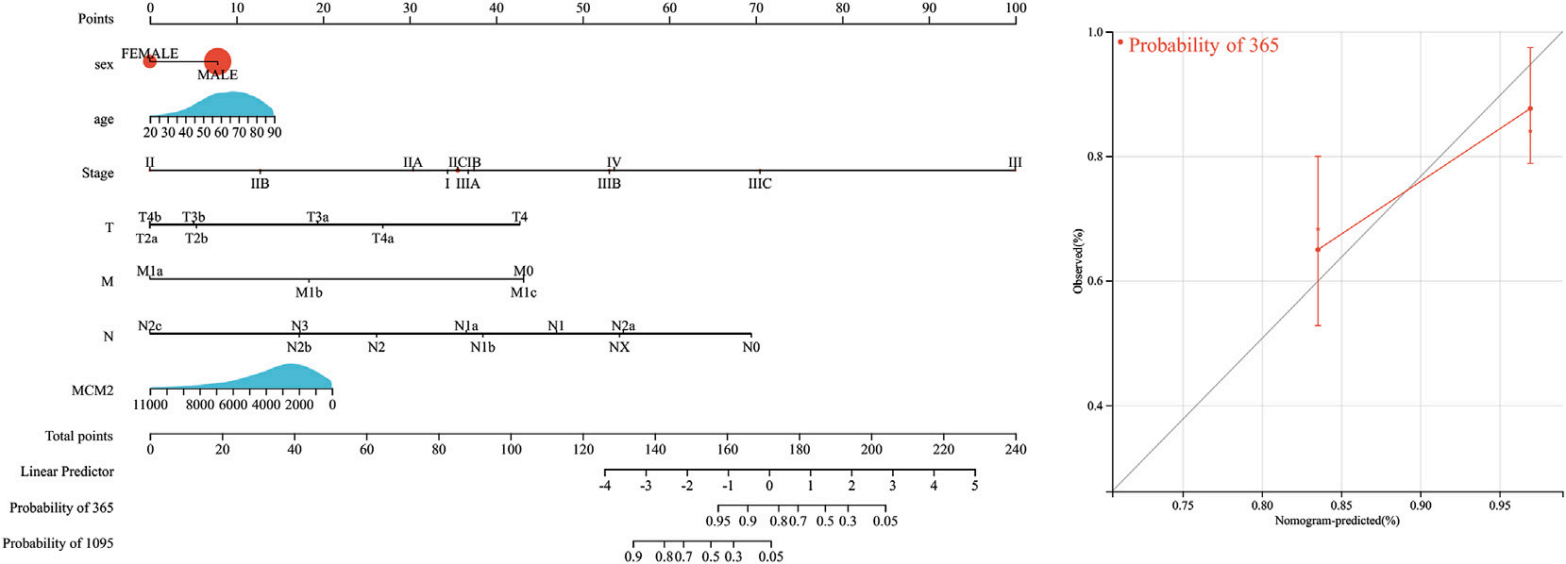
Survival nomogram and calibration curves. Prediction of 1-year and/or 3-year OS in SKCM patients.

We extracted the top 50 genes positively correlated with MCM2 that were co-expressed in SKCM from the STRING database in the form of a heatmap (Figure 11A). Then, we evaluated the correlation between MCM2 and the top 10 positively correlated genes. Our results demonstrated that MCM2 was strongly co-expressed with MCM5 (*r* = 0.607), ORC1 (*r* = 0.631), MCM6 (*r* = 0.544), MCM7 (*r* = 0.548), MCM10 (*r* = 0.515), and POLD1 (*r* = 0.51) (Figure 11B). To further understand the role of MCM2 in SKCM, GSEA was used to investigate the KEGG pathways involved in the difference between the MCM2-high and MCM2-low expression groups in SKCM. MCM2 was significantly enriched in pyrimidine metabolism (NES = −2.3019), purine metabolism (NES = −2.2391), base excision (NES = −2.2025), DNA replication (NES = −2.1527), progesterone-mediated oocyte maturation (NES = −2.1264), RNA polymerase (NES = −2.0594), the cell cycle (NES = −2.0808), nucleotide excision repair (NES = −2.0603), the pentose phosphate pathway (NES = −2.0210) and lysine degradation (NES = −2.0220) (Figure 11C). Next, univariate logistic regression analysis based on data from TCGA further determined the relationship between MCM2 expression in SKCM and clinicopathological variables, including cancer stage (T, N, M, or pathological stage), sex and age. Taking median MCM2 mRNA levels as the segmentation point, 235 patients were assigned to the low MCM2 expression group and 236 patients to the high MCM2 expression group. MCM2 expression was only significantly correlated with pathological stage (*p* = 0.022). However, significant correlation was not showed between MCM2 expression and T stage (*p* = 0.113), N stage (*p* = 0.116), M stage (*p* = 1.000), sex (*p* = 0.594) or age (*p* = 0.678) (Table 1). An established nomogram model was used to combine several clinical factors to analyze the ability of these clinicopathological variables to predict 1-year and 3-year OS in SKCM (Figure 12). The calibration curves for the probability of 1-year survival presented a high agreement with the nomogram-predicted probability (Figure 12).

### MCM2 is Upregulated in SKCM Cells and Promotes Cell Proliferation *In Vitro*

**FIGURE 13:**
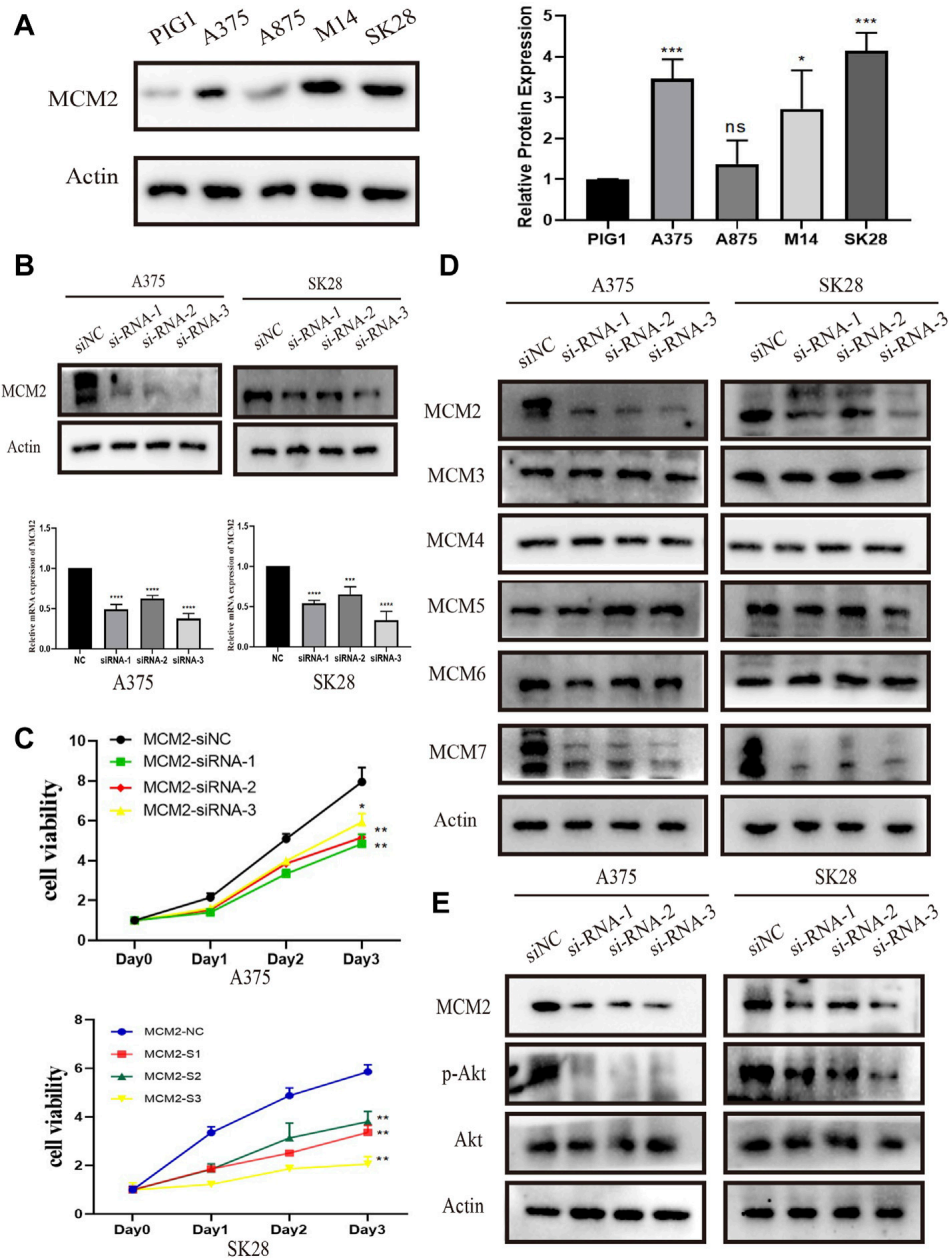
MCM2 is upregulated and promotes cell proliferation in SKCM *in vitro*. **(A)** The relative expression of MCM2 in SKCM cell lines (compared with that of actin) examined *via* Western blotting. **(B)** MCM2 was successfully downregulated in the A375 and SK28 cell lines in protein and transcriptional level by siRNA. **(C)** The MCM2 knockdown significantly suppressed cell proliferation by CCK8 assay. **(D)** The side-effect of MCM2 knockdown to other MCM proteins. **(E)** The MCM2 knockdown significantly suppressed the phosphorylation of Akt.

To further verify the results of the analysis above, MCM2 protein levels were examined in SKCM cell lines. The protein levels of MCM2 were significantly overexpressed in the cancer cell lines A375, A875, M14 and SK28 compared to the normal human skin melanocyte line PIG1, indicating that MCM2 was extremely overexpressed in A375 and SK28 cells. Thus, A375 and SK28 cells was used for the next experiments (Figure 13A). To investigate the potential role of MCM2 in SKCM, MCM2 was significantly knocked down in A375 and SK28 cells (Figure 13B). According to the database mining results and published literatures, MCM2 mainly participates in DNA replication and cell cycle division; thus, we performed a CCK-8 assay to evaluate the effect of MCM2 expression on cellular proliferation and Western blotting to verify the expression of proliferation-related signaling pathways. The CCK-8 assay showed that the cell viability of the MCM2-knockdown group was markedly decreased compared to that of the negative control (NC) group (Figure 13C). We further examined the side-effect of MCM2 knockdown to subunit, including MCM3-7. We found that MCM7 significantly downregulated in the MCM2 knockdown cells, while MCM3-6 did not show any changes (Figure 13D). Moreover, downregulation of p-Akt was detected in MCM2-knockdown cells (Figure 13E). These results indicated that MCM2 was upregulated in SKCM and promoted cell proliferation *in vitro* by affecting MCM7 expression and activating the Akt pathways.

## Discussion

Cancer cells are characterized by unlimited replication potential (Hanahan and Weinberg, 2011). MCM2 belongs to a protein family that is an important component in DNA replication licensing complexes (Liu et al., 2021). Cumulative evidence suggests that MCM2 proteins play a crucial role in maintaining the malignancy of cancer cells by interacting with several proteins. For example, nuclear factor κB (NF-κB), a transcription factor that has been demonstrated in multiple solid and hematological cancers, has been reported to regulate MCM2 to maintain the stem cell-like properties of colon cancer cells (Wang et al., 2020). Recently, MCM2 has been reported to be overexpressed in various cancer tissues (Ladstein et al., 2010; Nodin et al., 2012; de Andrade et al., 2013). However, the clinical value of MCM2 proteins across cancers remains unclear. Here, we explored the role of MCM2 in cancer diagnosis, therapy and prognosis, as well as its interaction with other genes and proteins and immune infiltration across cancers.

The differential expression of MCM2 has been reported in many cancers, but a comprehensive pan-cancer analysis is lacking. In the current study, we analyzed the expression and sublocalization of MCM2 in 33 cancers and found that MCM2 was significantly upregulated in 30 cancers and that mainly located in the nucleus of cells. In addition, MCM2 expression was significantly correlated with molecular and immune cancer subtypes and this is the first study to analyze this relationship.

We also investigated the gene expression characteristics of MCM2 which presented a high mutation rate, indicating that MCM2 may be a potential marker for diagnosing various cancers. We found the top-10 gene that was co-mutated with MCM2, which have been reported in multiple cancers, such as colorectal cancer (Li et al., 2022), BRCA (Lin et al., 2020), LUAD (Zhang et al., 2020) and LIHC (Li et al., 2021a). Furthermore, we integrated MCM2 in the GGI and PPI networks and found that it was strongly associated with genes and proteins related to the cell cycle and DNA replication, such as the CDC family, a group of genes coding proteases and phosphatases that regulate the cell division phase and cell cycle, and POLA1, a protein contributing to the DNA replication pathway. MCM2 is the most researched protein in cancers. In SKCM, MCM2 was highly overexpressed in tissues (de Andrade et al., 2013; Aihemaiti et al., 2018). Shaimaa et al. reported that MCM2 was an important downstream target of CDC7, as CDC7 significantly improved the chemoresistance of SKCM to BRAF^V600E^-specific inhibitors, indicating that MCM2 may be a potential therapeutic target for cancers (Gad et al., 2019). MCM2 was reported to be expressed more frequently than the published marker Ki-67, indicating that it could be a promising independent prognostic marker in breast cancer (Gonzalez et al., 2003; Issac et al., 2019). In addition, MCM2 increased the sensitivity of ovarian cancer to carboplatin therapy by affecting the expression level of cell cycle-related factors, such as p53 (Deng et al., 2019). In this study, drug susceptibility analysis showed that MCM2 expression was positively correlated with nine drugs, and negatively correlated with 27 drugs. Therefore, this provided additional targets for cancer chemotherapy.

Moreover, we found that MCM2 expression is strongly related with immune cell infiltration and immune-related molecule expression in most cancers, indicating that MCM2 may be a promising biomarker for immune therapy. An AUC analysis revealed that MCM2 had better predictive power than other some of the published biomarkers in estimating immune therapy outcomes. Immune checkpoint inhibitors (ICIs) have propelled immune therapeutics for cancer treatment but are only beneficial to some patients, which means that it is urgent to search for novel prognostic and predictive biomarkers for immune therapy, such as ICI treatment in cancers. Some previous studies have been reported on MCM2 in different cancers. For instance, MCM2 was also overexpressed in human tissues and associated with overall survival in SCLC. Moreover, highly expressed MCM2 was correlated with increased resistance to not only cisplatin but also anti-PD-1 treatment (Gao et al., 2021).

Next, we focused on the effect of MCM2 in SKCM. We integrated MCM2 with the top 50 positive co-expressed partners in SKCM and found strong correlations with the level of mRNA expression. GSEA also determined the top 10 signaling pathways in which MCM2 may play a role in regulating SKCM development. Consistent with our pan-cancer analysis findings that MCM2 is a potential biomarker for cancer diagnosis and prognosis, our results also indicated that MCM2 is an independent prognostic gene for SKCM, as assessed by a nomogram model according to baseline clinical data. Interestingly, previous studies have also reported the potential for MCM2 to predict SKCM using subtractive hybridization techniques. Spanjaard et al. (1997) detected several differentially expressed cDNAs that are associated with retinoic acid (RA)-induced growth arrest in SKCM. One of the strongly downregulated genes was MCM2, which was observed to have a potential role in promoting cancer cell growth in RA-resistant cells.

In addition, upon further exploration of the critical role of MCM2 in promoting cancer progression, we performed cell and molecular experiments to verify the associations between MCM2 expression and the activities of SKCM cell lines. As predicted, we found that the expression levels of MCM2 were much higher in cancer cells than in normal cell lines and that downregulating MCM2 in SKCM cells significantly inhibited cell proliferation. Interestingly, we found that MCM7 was significantly downregulated in MCM2-knockdown groups, indicating the MCM7 may be essential link for MCM2 promoting melanoma cell proliferation. According to previous study, MCM7 has also been reported as a promising target for different cancer types, such as liver cancer (Su, 2022), acute myeloid leukemia (Zhang et al., 2021b) and COAD (Li et al., 2021b). Surprisingly, lower MCM2 expression levels were associated with suppression of the Akt signaling pathways, which was consistent with our findings that these pathways were enriched by MCM2 in SKCM. Akt is central nodes of many signaling pathways and modulate many downstream molecules involved in cellular proliferation, survival and metabolism (Santos and Crespo, 2018; Revathidevi and Munirajan, 2019). The mechanism underlying the regulatory mechanism of the expression levels of Akt merits further investigation. Collectively, our study revealed that MCM2 is a promising biomarker for cancer diagnosis, therapy design and prognosis and follow-up. To our delight, though the experiment in melanoma cells, we verified that MCM2 promote cell proliferation *via* regulating the Akt signaling pathway *in vitro.*

In summary, the results of the multivariate analysis provide a systematic and comprehensive review of the biological characteristics of MCM2 across cancers and revealed that MCM2 might be a promising biomarker for cancer diagnosis and prognosis. Because of the correlation between MCM2 and various immune cells and molecules, we explored the potential relationship between MCM2 and immune cell infiltration and the predictive power of MCM2 in responses to immune therapy, indicating that MCM2 may be involved in regulation of immune infiltration and may be a potential biomarker for immune therapy. In conclusion, MCM2 plays a pivotal role in immunotherapy of the TME, prognoses and therapeutic response across all TCGA cancers by affecting infiltration of immune cells. More interestingly, our analysis also emphasized that MCM2 may be a vital protein which promote proliferation of SKCM and could serve as a therapeutic target.

## Data Availability

The datasets presented in this study can be found in online repositories. The names of the repository/repositories and accession number(s) can be found in the article/Supplementary Material.

## Supplementary Material

Supplementary Figure S1 The somatic mutations of MCM2 in various cancers. Pie chart showing the percentage of different MCM2mutations in the breast, cervix, kidney, liver, lung, prostate, skin, stomach, and thyroid according to the COSMIC database.

## Glossary

MCM2: Minichromosome maintenance 2
TCGA: The Cancer Genome Atlas
pre-RC: prereplicative complex)
ORC: origin recognition complex
HR: homologous recombination
PPI: protein–protein interaction
HPA: Human Protein Atlas
TMB: tumor mutation burden
COSMIC: catalog of somatic mutations in cancer
TIMER: Tumor Immune Estimation Resource
TIDE: Tumor Immune Dysfunction and Exclusion
GGI: gene-gene interaction
STRING: Search Tool for the Retrieval of Interacting Genes
GO: gene ontology
KEGG: Kyoto Encyclopedia of Genes and Genes
GSEA: gene set enrichment analysis
CPTAC: Clinical Proteomic Tumor Analysis Consortium
OS: overall survival analysis
UCSC: University of California Santa Cruz
ROC: receiver operating characteristic
AUC: areas under the curve
ATCC: American Type Tissue Culture Collection
ER: endoplasmic reticulum
FUCCI: Fluorescent Ubiquitination-based Cell Cycle Indicator
MSIsensor: microsatellite instability sensor
MSI-H: microsatellite instability high
MSI-L: microsatellite instability low
MSS: microsatellite stable
MMR: mismatch repair
ICGs: immune checkpoint genes
NF-κB: nuclear factor κB
ICIs: Immune checkpoint inhibitors
ACC: adrenocortical carcinoma
BLCA: bladder urothelial carcinoma
BRCA: breast invasive carcinoma
CESC: cervical squamous cell carcinoma and endocervical adenocarcinoma
CHOL: cholangiocarcinoma
COAD: colon adenocarcinoma
DLBC: lymphoid neoplasm diffuse large B-cell lymphoma
ESCA: esophageal carcinoma
GBM: glioblastoma multiforme
HNSC: head and neck squamous cell carcinoma
KICH: kidney chromophobe
KIRC: kidney renal clear cell carcinoma
KIRP: kidney renal papillary cell carcinoma
LAML: acute myeloid leukemia
LGG: brain lower‐grade glioma
LIHC: liver hepatocellular carcinoma
LUAD: lung adenocarcinoma
LUSC: lung squamous cell carcinoma
MESO: mesothelioma
OV: ovarian serous cystadenocarcinoma
PAAD: pancreatic adenocarcinoma
PCPG: pheochromocytoma and paraganglioma
PRAD: prostate adenocarcinoma
READ: rectum adenocarcinoma
SARC: sarcoma
SKCM: skin cutaneous melanoma
STAD: stomach adenocarcinoma
TGCT: testicular germ cell tumors
THCA: thyroid carcinoma
THYM: thymoma
UCEC: uterine corpus endometrial carcinoma
UCS: uterine carcinosarcoma
UVM: uveal melanoma
CIN: chromosomal instability
GS: genomically stable
POLE: Polymerase ε
EBV: Epstein-Barr virus
