# Supplementary material for: Multi-Omics Analysis of MCM2 as a Promising Biomarker in Pan-Cancer
Source: Front Cell Dev Biol. 2022 May 25;10:852135. doi: 10.3389/fcell.2022.852135 (PMC9174984; doi:10.3389/fcell.2022.852135)
Supplement: Supplementary file 1 [file Table1.docx]

| cancer | Purity | B Cell | CD4+ T Cell | CD8+ T Cell | Dendritic Cell | Macrophage | Neutrophil |
| --- | --- | --- | --- | --- | --- | --- | --- |
| ACC | 0.189128 | 1.22E-05 | 0.609742 | 0.368684 | 2.02E-05 | 0.099396 | 0.007708 |
| BLCA | 0.873887 | 0.091263 | 0.820772 | 1.44E-09 | 6.67E-10 | 3.22E-05 | 1.43E-06 |
| BRCA | 3.17E-13 | 4.06E-11 | 3.07E-05 | 0.210591 | 2.42E-08 | 0.104707 | 2.47E-07 |
| BRCA-Basal | 0.057542 | 0.342526 | 0.020374 | 0.935252 | 0.021684 | 0.229039 | 0.087359 |
| BRCA-Her2 | 0.325728 | 0.181064 | 0.261645 | 0.773504 | 0.678951 | 0.764911 | 0.883953 |
| BRCA-Luminal | 1.24E-12 | 0.011683 | 0.010014 | 0.152967 | 0.000569 | 0.266976 | 0.001857 |
| CESC | 0.078744 | 0.128323 | 0.004955 | 0.669154 | 0.123438 | 0.442309 | 0.016656 |
| CHOL | 0.411334 | 0.59002 | 0.966229 | 0.291077 | 0.531436 | 0.831015 | 0.911163 |
| COAD | 0.126345 | 0.751389 | 0.001563 | 0.154201 | 0.001135 | 0.097812 | 2.75E-05 |
| DLBC | 0.320441 | 0.057172 | 0.404786 | 0.785384 | 0.275616 | 0.532983 | 0.571662 |
| ESCA | 0.000236 | 0.260878 | 0.436452 | 0.084244 | 0.046301 | 0.624306 | 0.049102 |
| GBM | 2.69E-21 | 0.163007 | 0.861793 | 0.378243 | 0.016286 | 0.278197 | 0.977386 |
| HNSC | 1.08E-10 | 2.01E-05 | 5.68E-10 | 0.011743 | 3.36E-05 | 5.90E-05 | 0.000747 |
| HNSC-HPVpos | 0.021339 | 0.051134 | 0.029141 | 0.032547 | 0.09398 | 0.797946 | 0.06085 |
| HNSC-HPVneg | 1.92E-06 | 0.198434 | 7.36E-08 | 0.809538 | 0.017224 | 0.000491 | 0.052328 |
| KICH | 0.331905 | 0.936193 | 0.49108 | 0.230387 | 0.822353 | 0.074914 | 0.06138 |
| KIRC | 0.014639 | 6.11E-11 | 2.79E-06 | 0.011344 | 1.47E-14 | 1.49E-08 | 1.59E-10 |
| KIRP | 0.017175 | 0.512039 | 0.18161 | 0.503447 | 0.257127 | 0.663191 | 0.79174 |
| LGG | 1.91E-05 | 3.95E-12 | 4.18E-05 | 3.41E-09 | 1.31E-10 | 3.01E-06 | 8.29E-08 |
| LIHC | 7.98E-05 | 4.09E-19 | 1.28E-09 | 3.48E-11 | 1.92E-22 | 2.28E-18 | 9.08E-14 |
| LUAD | 0.514477 | 0.229728 | 0.632825 | 0.024321 | 0.016986 | 0.717936 | 2.42E-06 |
| LUSC | 1.73E-10 | 0.014487 | 0.061883 | 0.305861 | 0.744717 | 0.032857 | 0.251694 |
| MESO | 0.337527 | 9.42E-05 | 0.057366 | 0.993243 | 0.000343 | 0.482037 | 0.000162 |
| OV | 1.12E-05 | 0.049783 | 0.001833 | 0.586553 | 0.002207 | 0.024757 | 0.050093 |
| PAAD | 0.670614 | 0.00855 | 0.093456 | 0.019718 | 2.07E-05 | 0.174216 | 0.035346 |
| PCPG | 0.043039 | 0.002188 | 0.088397 | 0.148678 | 0.540865 | 0.016131 | 0.07883 |
| PRAD | 0.093336 | 4.58E-17 | 0.021381 | 1.97E-13 | 2.21E-09 | 4.66E-10 | 8.66E-13 |
| READ | 0.560464 | 0.236289 | 0.15405 | 0.157191 | 0.11922 | 0.10423 | 0.681258 |
| SARC | 1.42E-08 | 0.038265 | 5.52E-05 | 0.136272 | 0.925838 | 0.001659 | 0.234876 |
| SKCM | 0.023024 | 0.000293 | 0.005812 | 0.002669 | 7.07E-08 | 0.599733 | 0.00037 |
| SKCM-Primary | 0.095352 | 0.005751 | 0.283577 | 0.066576 | 0.061087 | 0.605864 | 0.129937 |
| SKCM-Metastasis | 0.098816 | 0.055073 | 0.088108 | 0.143395 | 8.45E-05 | 0.507579 | 0.044235 |
| STAD | 0.043685 | 3.74E-06 | 0.017606 | 0.483846 | 0.177305 | 2.25E-09 | 0.764867 |
| TGCT | 0.008451 | 0.532179 | 6.63E-08 | 0.004243 | 0.310998 | 0.243723 | 0.000775 |
| THCA | 0.128221 | 5.20E-48 | 1.93E-22 | 2.95E-05 | 5.17E-17 | 1.02E-25 | 3.38E-10 |
| THYM | 0.321855 | 7.76E-25 | 2.57E-08 | 1.19E-09 | 3.42E-15 | 1.42E-10 | 0.039373 |
| UCEC | 0.299621 | 0.028101 | 0.307349 | 0.131439 | 0.262448 | 0.016664 | 6.98E-05 |
| UCS | 0.56497 | 0.75848 | 0.704915 | 0.733137 | 0.636437 | 0.524933 | 0.179836 |
| UVM | 0.083412 | 0.398549 | 0.739409 | 0.132192 | 0.231561 | 0.10021 | 0.001902 |

Table S1: p value of the correlation between cancers and immune cells.
