# Supplementary figures and images for: Multi-Omics Analysis of MCM2 as a Promising Biomarker in Pan-Cancer
Source: Front Cell Dev Biol. 2022 May 25;10:852135. doi: 10.3389/fcell.2022.852135 (PMC9174984; doi:10.3389/fcell.2022.852135)

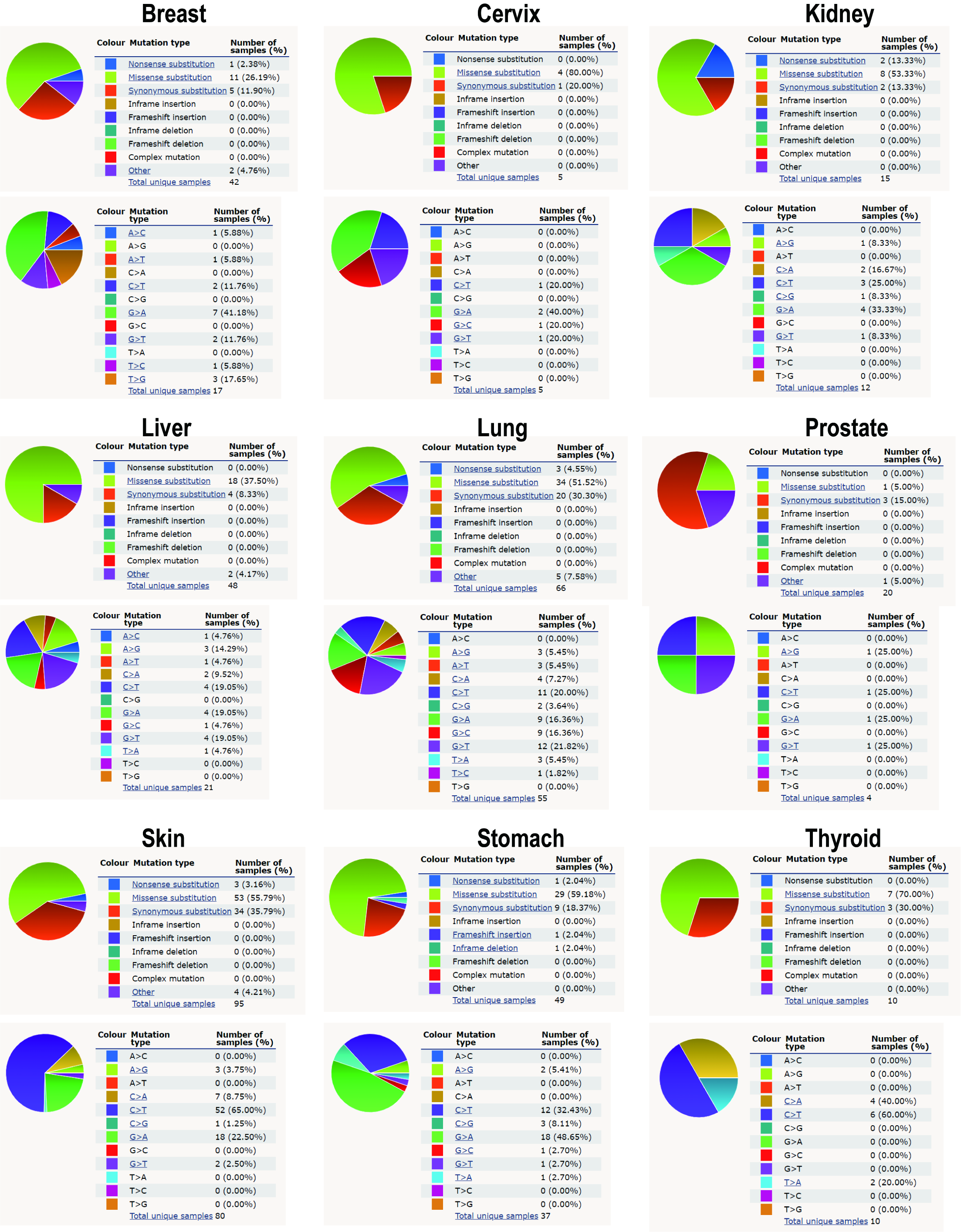

Supplement: Supplementary file 2 [file Image1.TIF]
